# Genomic insights into virulence mechanisms of *Leishmania donovani*: evidence from an atypical strain

**DOI:** 10.1186/s12864-018-5271-z

**Published:** 2018-11-28

**Authors:** Sumudu R. Samarasinghe, Nilakshi Samaranayake, Udeshika L. Kariyawasam, Yamuna D. Siriwardana, Hideo Imamura, Nadira D. Karunaweera

**Affiliations:** 1 Department of Parasitology, Faculty of Medicine, University of Colombo, Colombo, Sri Lanka; 2 Department of Biomedical Sciences, Institute of Tropical Medicine, Antwerp, Belgium

**Keywords:** Cutaneous leishmaniasis, High throughput sequencing, Aneuploidy, Copy number variations, SNPs, Drug resistance, Tropism, Indian subcontinent, Virulence genes

## Abstract

**Background:**

Leishmaniasis is a neglected tropical disease with diverse clinical phenotypes, determined by parasite, host and vector interactions. Despite the advances in molecular biology and the availability of more *Leishmania* genome references in recent years, the association between parasite species and distinct clinical phenotypes remains poorly understood. We present a genomic comparison of an atypical variant of *Leishmania donovani* from a South Asian focus, where it mostly causes cutaneous form of leishmaniasis.

**Results:**

Clinical isolates from six cutaneous leishmaniasis patients (CL-SL); 2 of whom were poor responders to antimony (CL-PR), and two visceral leishmaniasis patients (VL-SL) were sequenced on an Illumina MiSeq platform. Chromosome aneuploidy was observed in both groups but was more frequent in CL-SL. 248 genes differed by 2 fold or more in copy number among the two groups. Genes involved in amino acid use (LdBPK_271940) and energy metabolism (LdBPK_271950), predominated the VL-SL group with the same distribution pattern reflected in gene tandem arrays. Genes encoding amastins were present in higher copy numbers in VL-SL and CL-PR as well as being among predicted pseudogenes in CL-SL. Both chromosome and SNP profiles showed CL-SL and VL-SL to form two distinct groups. While expected heterozygosity was much higher in VL-SL, SNP allele frequency patterns did not suggest potential recent recombination breakpoints. The SNP/indel profile obtained using the more recently generated PacBio sequence did not vary markedly from that based on the standard LdBPK282A1 reference. Several genes previously associated with resistance to antimonials were observed in higher copy numbers in the analysis of CL-PR. H-locus amplification was seen in one cutaneous isolate which however did not belong to the CL-PR group.

**Conclusions:**

The data presented suggests that intra species variations at chromosome and gene level are more likely to influence differences in tropism as well as response to treatment, and contributes to greater understanding of parasite molecular mechanisms underpinning these differences. These findings should be substantiated with a larger sample number and expression/functional studies.

**Electronic supplementary material:**

The online version of this article (10.1186/s12864-018-5271-z) contains supplementary material, which is available to authorized users.

## Background

Leishmaniasis is a neglected tropical disease with spread across 98 countries over five continents. Constituting a spectrum of diseases ranging from fatal visceral disease to localized cutaneous disease, around 350 million of the global population is at risk of developing leishmaniasis in one of its many forms [1]. Leishmaniasis is caused by over 20 species of *Leishmania* parasites via sandfly bites. Moreover, the same parasite species has been known to cause different clinical phenotypes. This complexity of the disease poses many challenges to control efforts which are further compounded by variable responses to drugs, not only across continents but even within the same region. While the resulting phenotype is influenced by the interactions between the parasite, susceptible host and vector as well as the environment, a more severe degree of pathogenicity such as involvement of viscera and drug resistance are considered to be virulent features largely determined by parasite genotypes.

A key approach to explore the variability of the parasite characteristics is through the study of its genome. The genomes of *Leishmania* vary from 29 Mb to 33 Mb in size and are organized into a variable number of chromosomes (i.e., 34 or 35 in new world species and 36 in old world species) [2]. The number of protein coding genes range from 8023 to 8412 [3]. However, species-specific genes per se have not provided an adequate basis to explain the wide phenotypic variability that is observed, with only a limited number of genes being differentially distributed [4, 5]. Trypanosomatid genomes have been shown to have a peculiar arrangement amongst eukaryotes, where genes are arranged into large polycistronic gene clusters and lack conventional transcription control factors such as RNA polymerase 11 promoters. Thus, at genomic level, other mechanisms of altering gene expression such as copy number variations of chromosomes and genes have been investigated to determine parasite genetic basis to the different attributes. Single nucleotide polymorphisms (SNPs) in genes encoding vital structural and functional proteins may represent another level of such adaptations.

The advent of high throughput sequencing technologies coupled with the availability of reference genome sequences for several *Leishmania* species allow detailed comparative analyses at both inter and intra species level in the quest to understand these differences. The ability to cause potentially fatal visceral disease and limited treatment options has made *Leishmania donovani* the focus of several studies attempting to elucidate genetic factors contributing to tropism and drug resistance of this parasite. A comparative analysis of multiple isolates of *L. donovani* from patients with visceral leishmaniasis (VL) from India and Nepal showed a low level of intra species SNP variation but extensive variation in copy number in chromosomes [6]. The same study suggested a change in copy number of genes in tandem arrays and an amplicon to be associated with drug resistance. A larger study which analyzed intra species variation of *L. donovani* from a similar group of patients showed a genetically distinct population of parasites frequently resistant to antimonials to have a two base-pair insertion in the aquaglyceroporin (*LdAQP1*) gene [7].

Sri Lanka is endemic for leishmaniasis and is faced with an unusual situation where a species which causes VL in many parts of the Indian sub-continent, i.e. *L. donovani*, causes localized cutaneous leishmaniasis (CL) [8]. Even though a few cases of visceral disease and mucosal localization have been reported [9], the prevalent clinical type in the island continues to be almost exclusively the cutaneous form [10]. While intra-lesional or intra-muscular sodium stibogluconate (SSG) is the accepted standard therapy in the local setting, delayed response or failure to respond to treatment is not uncommon [11].

The causative parasite of both CL and VL in Sri Lanka, *Leishmania donovani* MON 37, has been shown to be genetically relatively close to *L. donovani* isolates from India, Bangladesh, and Nepal by both microsatellite and minicircle DNA analysis [12, 13]. A recent study comparing one isolate each from a CL and a VL patient suggested that SNPs and variable gene expression of *RagC* and *A2* genes are likely to be responsible for disease tropism [1]. Using next generation sequencing technologies, we describe here the genomic diversity of 8 clinical isolates of *L. donovani* from Sri Lanka; which included 2 isolates from patients diagnosed with VL and 6 isolates from patients diagnosed with CL. Among the 6 CL patients, 2 responded poorly to antimony treatment.

## Results

The six cutaneous leishmaniasis (CL-SL) and two visceral leishmaniasis (VL-SL) clinical isolates were from patients originating from different regions of the country (Additional file 1: Table S1-(A)). Raw sequencing read statistics for the parasite genomes are available in the Additional file 1: Table S1-(B).

### Genomic diversity in *L. donovani* isolates from CL and VL patients

#### Chromosome copy number/somy variations

**Fig. 1:**
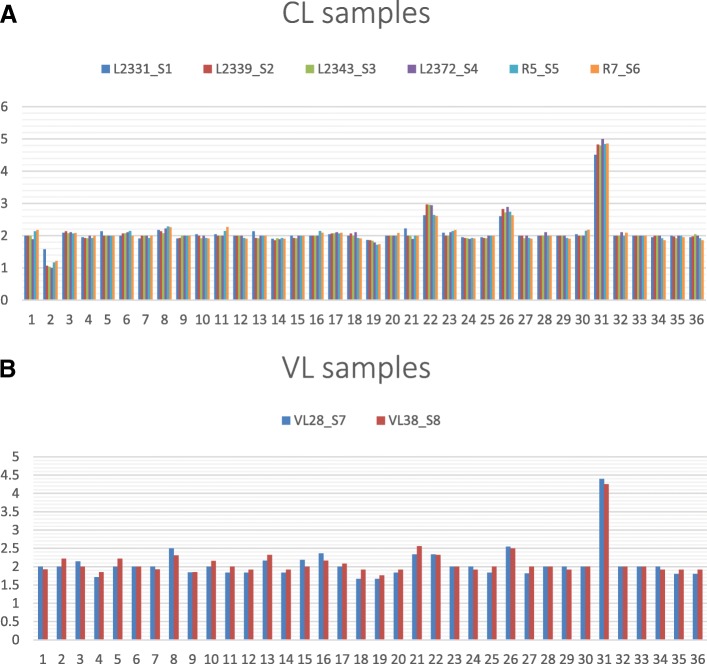
Chromosome somy distribution among Sri Lankan *L. donovani* isolates. **a** CL-SL isolates: Most chromosomes are disomic except for monosomic chromosome 2, trisomic chromosomes 22 and 26 and pentasomic chromosome 31 (**b**) VL-SL isolates: All chromosomes are disomic except for tetrasomic chromosome 31

We used normalized read depth data to determine copy number variations in chromosomes and protein-coding genes as described in Methods (Additional file 1: Table S2). Somy variations were seen in several chromosomes among the isolates (Additional file 1: Table S3). In CL-SL isolates the majority of chromosomes were disomic. Deviation from this was observed in chromosome 2 with monosomy, chromosome 22 and 26 with trisomy, and chromosome 31 with pentasomy (Fig. 1a). Overall, the chromosomes were disomic in VL-SL except for chromosome 31 which was tetrasomic (Fig. 1b). Furthermore, read depth changes suggestive of possible amplifications, affecting particular sub-regions of chromosomes (for example, chr2, chr8, chr12, chr22, chr33 and chr34; Additional file 2: Figure S1 and Additional file 3: Figure S2) were also observed in our isolates.

**Fig. 2:**
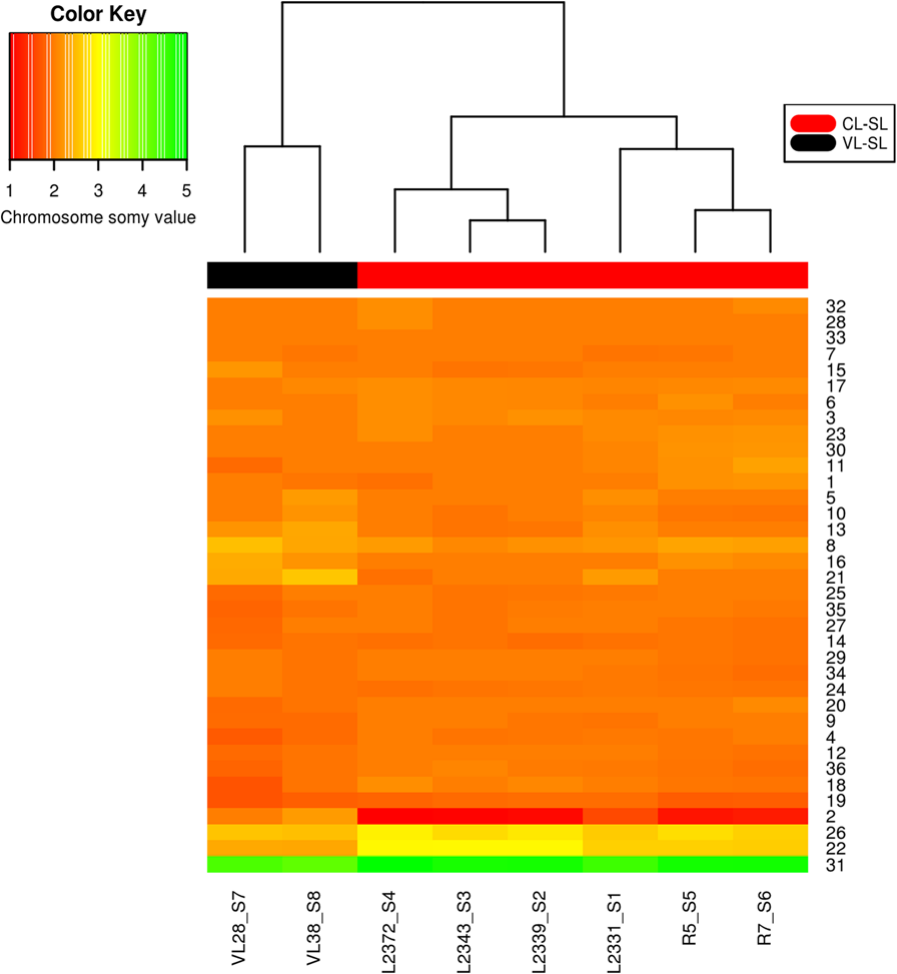
Heat map showing the comparison of aneuploidy profiles of CL-SL and VL-SL isolates. Chromosome numbers are listed along the right side y-axis and *L. donovani* CL and VL isolates are listed along the bottom x-axis. The dendrogram on top of heat map indicates the clustering pattern of the 8 isolates based on their aneuploidy similarity. The colour bar below the dendrogram represents the phenotype of isolate (Red-CL-SL and Black-VL-SL). The colour key represents the ploidy level of chromosomes in the heat map: chromosome somy > 4 green, trisomy – yellow, disomy – orange and monosomy – red

The heat map demonstrating the aneuploidy profiles of *Leishmania donovani* CL-SL and VL-SL isolates further confirmed the chromosome somy variations described above (Fig. 2). As indicated in the dendrogram, based on the similarity of aneuploidy profiles, VL-SL and CL-SL isolates clustered into separate groups. Three CL-SL isolates (L2339_S2, L2343_S3 and L2372_S4), showed high similarity to each other. CL-SL poor responders to SSG (R5_S5 and R7_S6) clustered with each other but separate from the other three CL-SL isolates mentioned earlier whereas L2331_S1, the remaining CL-SL isolate, was an exception and appeared to be more similar to the CL-SL poor drug responder group.

#### Gene copy number and tandem arrays

We identified 248 protein-coding genes in total with at least a 2-fold change in copy number between CL-SL and VL-SL (Additional file 1: Table S4). Genes with most varying copy numbers between *L. donovani* CL and VL isolates are summarized in Table 1.

**Table 1:** Genes with highly variable copy numbers between Sri Lankan *L. donovani* CL-SL and VL-SL isolates

Chr	GeneID	VL/CL Ratio	MWU-test p-value	Benjamini-Hochberg p-value	Product Description
27	LdBPK_271940	7.96	0.2717	0.3702	D-Lactate Dehydrogenase-Like Protein
27	LdBPK_271950	6.84	0.0012	**0.0029**	Branched-Chain Amino Acid Aminotransferase, Putative (Fragment)
10	LdBPK_100520	4.698	0.0000	**0.0000**	Gp63, Leishmanolysin
29	LdBPK_291600	4.41	0.0000	**0.0000**	Nodulin-Like, Putative
23	LdBPK_230360	4.22	0.0000	**0.0000**	Hypothetical Protein, Conserved
23	LdBPK_230350	3.86	0.0022	**0.0050**	Hypothetical Protein, Conserved
23	LdBPK_230330	3.85	0.0133	**0.0264**	Checkpoint Protein Hus1, Putative
25	LdBPK_210020	3.80	0.0009	**0.0023**	Histone H4
10	LdBPK_100920	3.55	0.8462	0.8817	Histone H3
23	LdBPK_230340	3.47	0.0001	**0.0004**	Tryptophanyl-Trna Synthetase, Putative
23	LdBPK_230320	3.06	0.2234	0.3163	Zinc Finger, C3Hc4 Type (Ring Finger), Putative
23	LdBPK_230310	3.02	0.7210	0.7877	Pteridine Reductase 1
11	LdBPK_110640	2.95	0.0922	0.1456	Metallo-Peptidase, Clan Mf, Family M17 (Fragment)
29	LdBPK_291590	2.90	0.3598	0.4576	Asparagine Synthase, Putative
10	LdBPK_100510	2.55	0.2285	0.3201	Gp63, Leishmanolysin (Fragment)
23	LdBPK_230290*	2.49	0.8027	0.8474	Abc-Thiol Transporter (Fragment)
23	LdBPK_230300	2.47	0.8735	0.9026	Argininosuccinate Synthase, Putative
23	LdBPK_230250*	2.47	0.0540	0.0905	Hypothetical Protein, Conserved
35	LdBPK_353690	2.38	0.0001	**0.0003**	Hypothetical Protein, Conserved
22	LdBPK_221370	2.36	0.0350	0.0616	40S Ribosomal Protein L14, Putative
31	LdBPK_310470	2.28	0.6628	0.7405	Hypothetical Protein
23	LdBPK_230370	2.27	0.0000	**0.0000**	Hypothetical Protein, Conserved
23	LdBPK_230260*	2.22	0.1946	0.2790	Hypothetical Protein, Conserved
25	LdBPK_020010	2.20	0.1855	0.2691	Aminopeptidase P1, Putative
15	LdBPK_150430	2.19	0.9484	0.9561	60S Acidic Ribosomal Protein, Putative
27	LdBPK_271490	2.13	0.1280	0.1947	Hypothetical Protein, Conserved
11	LdBPK_111070	2.13	0.3303	0.4289	Hypothetical Protein
28	LdBPK_282050	2.12	0.4786	0.5652	Zinc Transporter 3, Putative
34	LdBPK_341720	2.10	0.9113	0.9300	Amastin-Like Surface Protein, Putative (Fragment)

Benjamini-Hochberg critical value for a false discovery rate < 0.05 was considered significant and marked in bold

^*^Genes previously reported with high copy number variations between a cutaneous and a visceral isolate from Sri Lanka [14]

We also identified 274 multicopy genes (tandem gene arrays) with at least a 2-fold change in gene copy number between Sri Lankan *L. donovani* CL and VL isolates (Additional file 1: Table S5). Multicopy genes are defined as genes of more than one copy with the same OrthoMCL group ID which are present on the same chromosome. 194 and 241 tandem gene arrays were observed in CL-SL and VL-SL respectively (Additional file 1: Table S6 and Table S7). Conforming to differences observed at individual gene level, the set of protein-coding genes with highly variable copy numbers were seen to be also present in the tandem gene arrays with highly variable copy numbers between CL and VL isolates (Table 2).

**Table 2:** Tandem gene arrays with highly variable copy numbers between *L. donovani* CL-SL and VL-SL isolates

Chr	OrthoMCL ID	Product description	No of genes in ref	Haploid number CL	Haploid number VL	VL/CL ratio
27	OG5_127093	D-lactate dehydrogenase-like protein	1	4	33	7.96
27	OG5_126731	Branched-chain amino acid aminotransferase, putative	1	5	35	6.84
29	OG5_131290	Nodulin-like, putative	1	1	6	4.41
23	OG5_154577	Hypothetical protein, conserved	1	1	4	4.22
23	OG5_181194	Hypothetical protein, conserved	1	1	5	3.86
23	OG5_150800	Checkpoint protein Hus1	1	1	5	3.85
21	OG5_126575	Histone H4	1	1	2	3.80
23	OG5_127096	Tryptophanyl-trna synthetase, putative	1	1	5	3.47
23	OG5_180874	Zinc finger, C3Hc4 type (ring finger) containing protein, putative	1	1	4	3.06
23	OG5_133937	Pteridine reductase 1	1	1	4	3.02
10	OG5_126749	Major surface protease Gp63, putative	2	7	21	3.00
34	OG5_130729	Amastin-like surface protein-like protein	2	4	2	0.50
22	OG5_126823	Ribonucleoside-diphosphate reductase small chain, putative	3	2	1	0.50
12	OG5_136657	Protein of unknown function (Duf962), putative	2	2	1	0.50
8	OG5_132981	Amastin, putative	1	2	1	0.44

#### Functional characterization of differentially distributed genes and arrays

D-lactate dehydrogenase-like protein (LdBPK_271940, OG5_127093) and branched-chain amino acid aminotransferase (LdBPK_271950, OG5_126731) were present in significantly higher copy numbers in VL-SL group (Tables 2 and 3). D-lactate dehydrogenase-like protein is involved in energy metabolism of *Leishmania* and may relate to optimal growth and survival of parasites within the host. Unlike other *Trypanosoma* species, D-lactate dehydrogenase is present only in *Leishmania* and absence of this enzyme in humans has suggested this to be a candidate drug target in these parasites [15, 16]. The extensive copy number variation seen in the branched-chain amino acid aminotransferase gene may result in variable consumption of amino acids by parasites.

**Table 3:** Effects of SNPs and short indels identified in Sri Lankan *L. donovani* isolates on coding regions

Variant type	Common to VL & CL	Unique to CL	Unique to VL
Frame shift	52	15	31
Stop gained	21	3	7
Stop lost	8	2	4
Start lost	2	2	1
Stop retained	13	3	3
Non synonymous coding	12,046	1576	2804
Synonymous coding	12,181	1558	2507
Initiator codon variant	1	–	–
Disruptive inframe deletion	30	4	3
Disruptive inframe insertion	35	4	10

Other genes with higher copy numbers in VL-SL, included *GP63* gene which encodes a surface protein previously reported as an important virulence factor in *Leishmania*, favoring its rapid migration, internalization and survival in host macrophages [17, 18] whereas checkpoint protein Hus1 in *Leishmania* has been shown to help the parasite to cope with replicative stress [19]. Pteridine reductase 1 encoding gene is a component of the pteridine metabolic pathways, which are highly adapted among these parasites which are pteridine auxotrophs. Changes that disrupt this pathway have been found to lead to attenuation or loss of virulence, inhibiting parasite survival and growth in animal models [20–22].

**Fig. 3:**
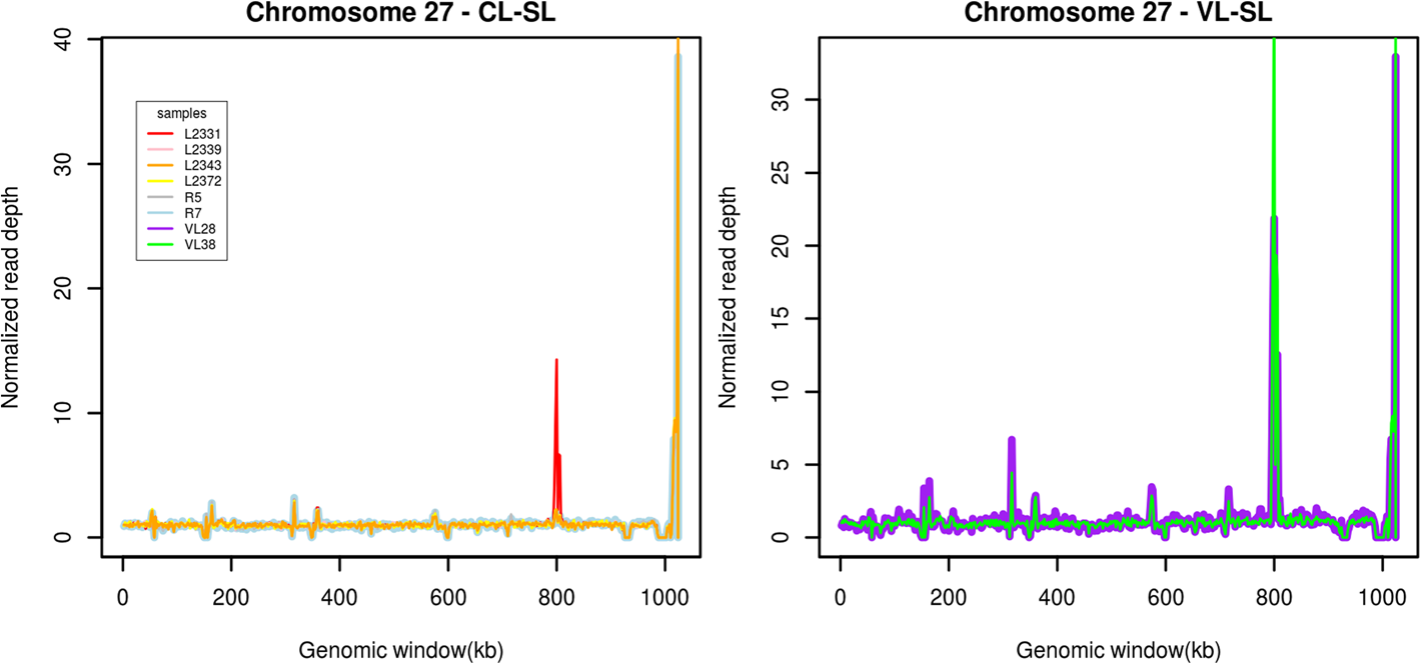
Genes with highly variable copy numbers on chromosome 27 among CL-SL and VL-SL isolates. LdBPK_271940 (Chr27: 798359–799,840) and LdBPK_271950 (Chr27: 803591–804,793). The two genes show a drastic increase in copy number among VL-SL isolates (VL28 and VL38) compared to CL-SL isolates (L2339, L2343, L2372, R5 and R7). As an exception L2331 CL isolate (red) shows a somewhat higher increase in copy number of the genes than other CL-SL isolates. Figure was generated using R graphics [73]

Several histone encoding genes were also present in a higher copy number in VL-SL isolates. Histones form the core of the parasite nucleosome and these structural proteins play an important role in DNA metabolism including transcription regulation, DNA repair and replication. These have also been identified as evolutionarily conserved proteins which act as prominent immunogens during *Leishmania* infections [23]. Distinct differences in protein biosynthesis and metabolism were suggested by markedly variable copy numbers of genes such as tryptophanyl-trna synthetase (LdBPK_230340, OG5_127096) which is also identified as important drug targets in eukaryotic parasites [24] and C3HC4 type zinc-finger (LdBPK_230320, OG5_180874) which has been shown to play a key role in the ubiquitination pathway modulating protein levels [25]. Since the *A2* gene locus on chromosome 22 is recognized to have misassembles in the *L. donovani* BPK282A1 reference [26], we analyzed the region using the more complete *L. donovani* PacBio sequence (Additional file 4: Figure S3) but did not observe a difference in copy number among CL-SL and VL-SL. Selected genes on chromosome 27 which demonstrated marked variability in copy number among the isolates are shown in Fig. 3.

#### GO enrichment analysis

**Fig. 4:**
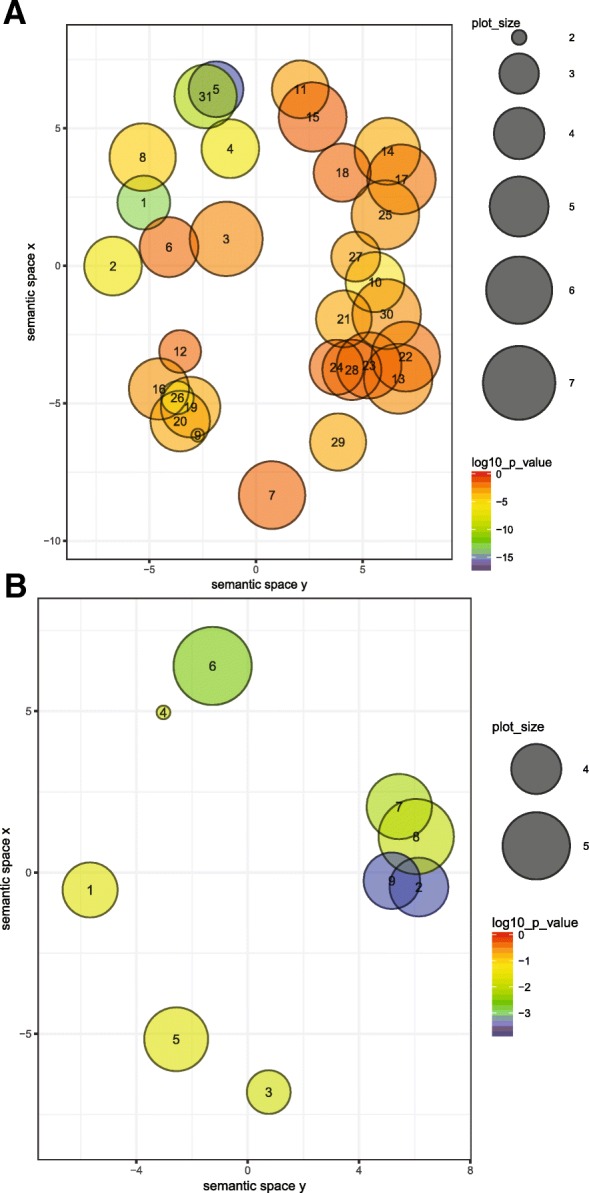
Enriched GO terms within the highly variable genes among CL-SL and VL-SL isolates. GO terms found to be overrepresented with a *p* < 0.05 are shown for biological processes in VL-SL (Panel **a**) and CL-SL (Panel **b**). A web-based tool, REVIGO was used to remove redundant GO terms among the gene set based on a chosen semantic similarity measure of 0.5. Remaining GO terms were represented as circles in a two dimensional scatter plot, where the size of the circles indicates the frequency of the GO term it represents and the distance between the circles indicates the semantic similarity between the corresponding GO terms. Circles closer to each other in the plot represent semantically similar GO terms. Circle color indicates the enriched GO term *p*-values according to the color key given in the plot. Number within each circle corresponds to the GO term as per Additional file 1: Table S9. Plot axes x and y is a general representative measure of semantic space

The genes that were differentially distributed between CL-SL and VL-SL isolates with at least a two-fold change in copy number (Additional file 1: Table S4) were analyzed for enrichment of GO terms (Additional file 1: Table S8 and Table S9). Genes identified with a higher copy number among the VL-SL group were associated with biological processes such as cell adhesion, biological adhesion, modulation by symbiont of host signal transduction pathway and macromolecular complex subunit organization, with these groups forming clear clusters (Fig. 4a). This is a likely reflection of the role of these processes in conferring parasite virulence features. The high copy number genes within the CL-SL group were associated with biological processes related to cellular response to oxidative stress, response to abiotic stimulus, response to chemicals and response to heat and proteolysis (Fig. 4b), indicating the cutaneous parasite’s adaptation to changes in the environment. Differences were also noted in the molecular functions of the two groups. High copy number genes in VL-SL were mainly associated with functions such as protein polymerization while peptidase activity was one of the main associations of the genes in the CL-SL group.

#### SNP/Indel diversity

A high quality set of SNPs and indels were obtained by site and genotype-level filtering of the raw SNP and indel set produced by GATK’s HaplotypeCaller as described in methods. We identified 85,642 variants (81,859 SNPs and 3783 indels) consistent within the 6 clinical samples of CL-SL against *L. donovani* reference. There were 69,841 variants (68,008 SNPs and 1833 indels) consistent within the 2 clinical samples of VL-SL against the *L. donovani* reference. Of these 56,560 SNPs and 1674 indels were common to isolates from the two phenotypes. 21,799 SNPs and 2109 indels were unique to CL-SL while 8688 SNPs and 159 indels were unique to VL-SL.

**Fig. 5:**
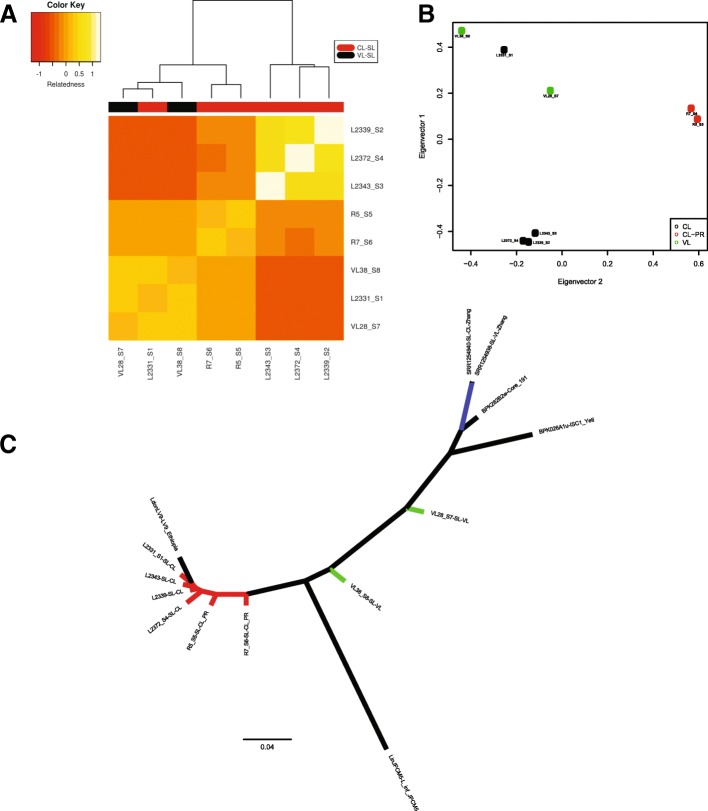
Relatedness and genetic divergence levels between the 8 Sri Lankan *L. donovani* isolates. **a** Heat map and associated dendrogram based on the similarity between the SNP profiles of 8 isolates. Each colored square in heat map represents the level of SNP similarity for an isolate listed along the y-axis compared to the isolate listed on the x-axis according to the colour key given. The colour bar below the dendrogram represents the phenotype of isolate: Red-CL-SL and Black-VL-SL. Three distinct clades are visible. CL-SL, VL-SL and CL-PR. L2331 CL isolate is an exception that it clusters with the VL group. **b** Principal component analysis based on a pruned SNP set of 8 isolates shows the same results as above (**c**) Phylogenetic tree based on maximum-likelihood method showing the genetic divergence levels between different *Leishmania* strains

Varying from the chromosome aneuploidy profiles, the heat map generated based on a similarity measure between the SNP profiles of 8 Sri Lankan isolates showed that they formed three distinct groups, which could also be related to their associated phenotype (Fig. 5a). Principal component analysis on the SNPs showed a similar population structure (Fig. 5b). Figure 5(c) represents the phylogenetic relationship between the 8 Sri Lankan *L. donovani* isolates and other key *Leishmania* strains described in a previous study [7]. Overall topology of the tree for the samples previously described by Imamura H and colleagues is similar to our current tree. This tree represents the genetic diversity of *L. donovani* in Sri Lanka that we examined, and is supported by many definitive homozygous SNPs. Although we still observed some base noises due to mismatched bases in the alignment, we have eliminated most of the potential computational errors such as missing information and low quality SNPs.

#### Functional variation of SNPs in protein-coding regions

Among the variants unique to CL-SL, 3169 SNPs were present in 1611 coding DNA sequences (CDS) and 34 indels in 27 CDS (Additional file 1: Table S10 and S11). Similarly, among the variants unique to VL-SL, we found 5357 SNPs in 1686 CDS and 54 indels in 43 CDS (Additional file 1: Table S12 and S13). The SNPs and their functional effects are summarized in Table 3.

Our results showed that 17 genes in CL-SL and 32 genes in VL-SL have been affected by frame shifts or stop codon variations unique to the isolates that could possibly give rise to pseudogenes and affect protein expression (Additional file 1: Table S14). Among the predicted pseudogenes in CL-SL isolates, some genes were identified with important putative functions. Amastin like surface proteins (LdBPK_044340) are abundant surface antigens which are known to be highly expressed in amastigote stage of *Leishmania* parasites. These are vital transporters in parasite survival inside host cells and are also signal transducers that allow the parasite to adapt to host environment [27]. Trypanothione reductase (LdBPK_050350), is a critically important enzyme for *Leishmania* parasite survival and their ability to survive inside macrophages can be significantly reduced due to partial loss of this protein activity [28] (Additional file 5: Figure S4). Similarly, the possible pseudogenes predicted among the VL-SL isolates include functionally important genes as well. Putative mitogen-activated protein kinases/*MAPKs* (LdBPK_020260, LdBPK_131380) are found to be vital components in *Leishmania* signaling cascades. Past studies have reported that inhibition of *MAPK*s activity relates to the increased survival of *L. donovani* parasites in human peripheral blood mononuclear macrophages [29].

Among the high SNP count genes in CL-SL and VL-SL, 685 and 734 genes respectively (Additional file 1: Table S15 and S16) were identified as genes under strong selection using SNP ratios as described in methods, possibly indicating the importance of these gene functions in *Leishmania* biology.

The expected heterozygosity (*He*) which is a measure for genetic diversity was higher in VL-SL isolates, with a negative inbreeding coefficient (Table 4). L2231 CL-SL isolate was more similar to VL-SL group with a higher number of heterozygous SNPs and a negative inbreeding coefficient. Among the other CL-SL isolates, the two isolates from poor responders to sodium stibogluconate (R5 and R7) appeared to be similar to each other. There were no distinct changes in allele profiles that were demonstrated by changes in heterozygous SNP coverage depth, and this indicated that there were no clear signs of potential recent recombination breakpoints on any of the chromosomes in the eight isolates even though losses of heterozygosity, where heterozygous SNPs were lost in a large block, were observed (Additional file 6: Figure S5).

**Table 4:** Heterozygosity and homozygosity within the CL-SL and VL-SL genomes

Isolate	H_o_	H_e_	Total SNPs	F (inbreeding coefficient)
VL38	26,663	57,592	84,255	−1.28637
L2331	38,248	58,153	96,401	−1.21113
VL28	13,098	24,524	47,622	−1.10192
R7	39,283	41,672	80,955	−0.77418
R5	44,411	40,095	84,506	−0.65821
L2343	56,029	6051	62,080	0.74412
L2339	57,733	4071	61,804	0.83690
L2372	52,717	2273	54,990	0.90426

Heterozygosity and homozygosity identified within the 8 isolate genomes, among a total of 100,318 SNPs. Number of heterozygous SNPs (H_e_), homozygous SNPs (H_o_) and inbreeding coefficient (F) were calculated using VCFtools

#### SNP/Indel analysis using a new genomic sequence of *L. donovani*

Refining existing *Leishmania* references using a PacBio sequencing platform have been reported in several *Leishmania* species, which improved both coverage and base accuracy of the repetitive regions of the current genome references [30, 31]. We used a more recently generated *L. donovani* genome sequence to complement our findings. The same protocol as described under methods was used to call variants. The results indicated that the SNP/indel counts generated using the more recent PacBio sequence and the standard LdBPK282A1 reference are compatible and did not vary markedly, partly because SNPs on repetitive regions with lower mapping quality were screened out (Table 5). A marginal increase in the total number of variants generated with the PacBio sequence is in agreement with the fact that it became slightly longer and has a lesser number of misassembles when compared to the standard LdBPK282A1 reference.

**Table 5:** Comparison of SNP/Indel profiling against different sequences of *L. donovani*

	SNP count		Indel count	
	LdBPK282A1 (Standard reference)	LdBPKPAC2016beta (PacBio sequence)	LdBPK282A1 (Standard reference)	LdBPKPAC2016beta (PacBio sequence)
Shared by both CL and VL	56,560	57,063	1674	1669
Unique to CL	21,799	24,858	2109	2140
Unique to VL	8688	7920	159	161

### Poor response to SSG among cutaneous isolates of *L. donovani*

#### Chromosome and gene copy number variations in comparison to drug response

**Fig. 6:**
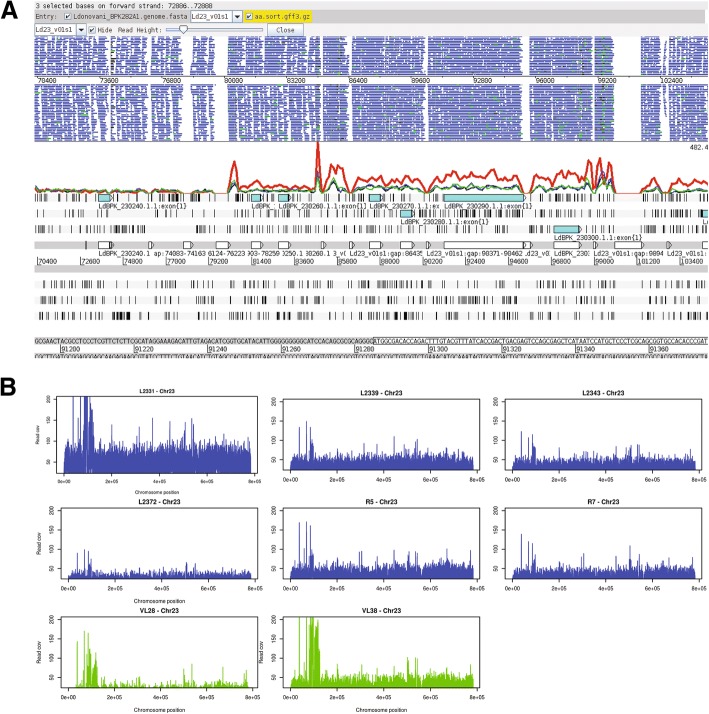
Comparison of read coverage across H-locus region of chromosome 23 among Sri Lankan *L. donovani* CL and VL isolates. **a** *L. donovani* H-locus region is defined between the coordinates 80,157 bp – 102,529 bp on chromosome 23. Few isolates were chosen randomly among CL and VL groups to clearly represent the difference in amplification of the H locus region; CL isolates - L2339 (Green), L2343 (Blue), R5 (Black) and VL isolate - VL38 (Red). Amplification of the H-locus region in VL-SL compared to CL-SL isolates were clearly observed in genes; Terbinafine resistance locus protein (LdBPK_230280: 89042–89,611), *MRPA* (LdBPK_230290: 91285–95,325) and Argininosuccinate synthase gene (LdBPK_230300: 96936–98,192). Figure was generated using Artemis tool **(b)** Read coverage across chromosome 23 in six CL-SL and two VL-SL *L. donovani* isolates. Amplification of the H-locus (80,157 bp – 102,529 bp) is clearly seen among VL-SL VL38 isolate (In VL28 the H-locus amplification was not prominent probably due to comparatively low coverage of the sample) and one CL-SL isolate was an exception (L2331). Figure was generated using R graphics

Chromosome copy numbers were not remarkably variable among the CL poor responders to SSG (CL-PR) and CL wild type (CL-WT) (Fig. 1a, Additional file 1: Table S3) in agreement with an earlier study that explains antimonial resistance in *L. donovani* clinical isolates [6]. Coverage analysis showed that H-locus region on chromosome 23 (Fig. 6), which includes ATP-binding cassette ABC transporter gene *MRPA* (LdBPK_230290), had notably amplified in our VL isolates. Among the six CL isolates this amplification was seen in only one isolate which however did not show a poor response to intra-lesional stibogluconate. Interestingly, the two CL isolates associated with a poor response to SSG in this study (R5 and R7) did not show a notable increase at the H-locus. The sensitivity of the two local VL isolates to stibogluconate is not known as the patients were treated with liposomal Amphotericin B as per local guidelines.

In addition to *MRPA*, H-locus comprises of another two important genes, terbinafine resistance locus protein (LdBPK_230280) and argininosuccinate synthase gene (LdBPK_230300). Terbinafine resistance locus protein has previously been identified to mediate efflux of terbinafine in *L. major* [32] and argininosuccinate synthase gene has been identified to be associated with antimony resistance in *L. infantum* [33]. Also an up regulation of argininosuccinate synthase gene was seen in *Leishmania* parasites as a response to oxidative stress and arginine starvation and the viability of the parasites were seen to notably reduce when the gene was down regulated, indicating an important role of the argininosuccinate synthase gene in infectivity and survival of the parasites in the mammalian host [34, 35].

We identified a total of 52 genes with at least a 2-fold change in gene copy number between CL-WT and CL-PR to SSG (Additional file 1: Table S17). However, only 27 genes showed a statistically significant change (FDR < 0.05 with Benjamini-Hochberg correction) between the two groups, where most of them were hypothetical proteins (Table 6). We identified an important gene encoding tryparedoxin peroxidase (LdBPK_151140) and also Cathepsin L-like protease with a relatively higher copy number in the CL-PR group, which was previously shown to have crucial roles related to antimony resistance in *L. donovani* and its survival as well as virulence [36]. Cysteine proteases (Cathepsin L- and B-like cysteine proteases) have been found to play an important role in pathogenesis and survival within the host macrophages in *Leishmania* and also have been identified as targets for drug design [37, 38].

**Table 6:** Genes with highly variable copy numbers between *L. donovani* CL-WT and CL-PR

Chr	GeneID	CL-PR/CL-WT ratio	MWU-test p-value	Benjamini-Hochberg p-value	Product Description
32	LdBPK_323700	3.42	0.0032	0.0087	Hypothetical Protein, Conserved
34	LdBPK_044270	2.35	0.1976	0.2635	Hypothetical Protein, Conserved
17	LdBPK_081310	2.26	0.4180	0.4932	Hypothetical Protein, Conserved
34	LdBPK_341300	1.86	0.0102	0.0221	Hypothetical Protein, Conserved
1	LdBPK_010160	1.83	0.0167	0.0347	Lsm Domain Containing Protein, Putative
32	LdBPK_322060	1.80	0.0245	0.0471	Hypothetical Protein
34	LdBPK_341720	1.80	0.0839	0.1364	Amastin-Like Surface Protein, Putative (Fragment)
12	LdBPK_120667	1.79	0.0000	0.0000	Hypothetical Protein (Fragment)
12	LdBPK_120210	1.70	0.1487	0.2092	Hypothetical Protein, Conserved
19	LdBPK_191070	1.69	0.0054	0.0134	Hypothetical Protein, Unknown Function
9	LdBPK_090180	1.68	0.4268	0.4932	Atg8/Aut7/Apg8/Paz2, Putative
21	LdBPK_211640	1.68	0.0000	0.0001	Zinc Finger Domain-Like Protein
30	LdBPK_301560	1.68	0.3125	0.3964	Hypothetical Protein
8	LdBPK_080950	1.67	0.0981	0.1545	Cathepsin L-Like Protease (Fragment)
15	LdBPK_151140	1.66	0.0000	0.0000	Tryparedoxin Peroxidase

We identified 186 tandem arrays in CL-WT (Additional file 1: Table S18) while there were 191 tandem arrays in CL-PR group (Additional file 1: Table S19). Interestingly, only 16 arrays had at least a 2-fold change in copy number between the two groups (Table 7, Additional file 1: Table S20). Among them quinonoid dihydropteridine reductase (QDPR) (OG5_131023), amastin surface glycoprotein (OG5_143904, LdBPK_341720.1) and histidine secretory acid phosphatase (OG5_158724) have shown an increase in its gene copy number in CL-PR group. QDPR has been characterized in *Leishmania major* and reported as a key enzyme required for regeneration and maintenance of H_4_biopterin, a molecule found to be crucial for growth, virulence and differentiation of *Leishmania* parasites [39].

**Table 7:** Tandem gene arrays with highly variable copy numbers between *L. donovani* CL-WT and CL-PR

Chr	OrthoMCL ID	Product description	Haploid CL-WT	Haploid CL-PR	CL-PR/CL-WT ratio
32	OG5_134607	Hypothetical protein, conserved	1	5	3.42
34	OG5_131023	Quinonoid dihydropteridine reductase	1	3	2.35
34	OG5_143904	Amastin surface glycoprotein, putative	3	5	1.80
12	OG5_145891	Hypothetical protein, conserved	1	2	1.70
21	OG5_136877	Palmitoyl acyltransferase 3, putative	1	2	1.68
32	OG5_157991	Hypothetical protein, conserved	1	2	1.65
32	OG5_166761	GIPL galf transferase, putative	1	2	1.65
36	OG5_184184	Hypothetical protein, conserved	1	2	1.62
35	OG5_204252	Hypothetical protein, conserved	1	2	1.59
4	OG5_148241	Hypothetical protein, conserved	2	3	1.58
36	OG5_126983	40S ribosomal protein S9, putative	1	2	1.57
36	OG5_158724	Histidine secretory acid phosphatase, putative	1	2	1.56
35	OG5_130002	Yos1-like, putative	1	2	1.51
36	OG5_127039	Sugar efflux transporter for intercellular exchange, putative	1	2	1.50
35	OG5_128319	ADP-ribosylation factor-like 2, arl2, putative	1	2	1.50
27	OG5_127093	D-lactate dehydrogenase-like protein	5	2	0.41

We observed a 2-fold increase in copy number of a tandem gene array encoding highly immunogenic amastin surface glycoprotein (OG5_143904, LdBPK_341720.1) in CL-PR group. *Leishmania* amastins are surface antigens predominantly expressed in amastigote stage of parasite’s life cycle. They are identified as essential transporters and signal transducers that help the parasites to adapt to changing environment within the host [27, 40]. A previous study has reported a gene which encodes an amastin (LdBPK_080760), with a significantly higher copy number in *Leishmania* parasites that have shown resistant to SSG in a clinical setting [6]. We observed the LdBPK_080760 gene within our previously discussed set of 52 genes with at least a 2-fold change in copy number (Additional file 1: Table S17). Hence we may argue that parasites use differential expression of amastins as a means of adapting to induced antimony pressure within host.

A tandem gene array (OG5_158724) consisting two gene types: LdBPK_366770 encoding a histidine secretory acid phosphatase and LdBPK_366740 encoding a tartrate-sensitive acid phosphatase showed a 2-fold increase in copy number among our poor responders to SSG (Table 7). Both these genes have been previously reported with a significantly higher copy number in a set of SSG resistant clinical isolates [6].

#### SNP/Indel distribution in comparison to drug response

We detected a total of 15,076 SNPs distributed in 3385 protein coding genes among the CL- PR group, of which 7361 were non-synonymous and 7665 were synonymous mutations. Genes annotated with high counts of non-synonymous SNPs mostly encoded hypothetical proteins. In addition, a phosphatidylinositol 3-kinase-like protein, a putative ABC1 transporter, putative Vps51/Vps67 and a putative glycosyl transferase were detected (Additional file 1: Table S21). Phosphatidylinositol 3-kinase-like protein and Vps51/Vps67 genes were annotated with high counts of non-synonymous SNPs among our CL-PR group. A non-synonymous coding SNP (Chromosome 2, position 36,464) in phosphatidylinositol 3-kinase (LdBPK_020100) has previously been identified to be highly differentiated between SSG resistant and susceptible lines [6]. Vps51/Vps67 domain as well as phosphatidylinositol 3-kinase domain have been found to be associated with TOR signaling pathway in *Trypanosomes* [41] possibly indicating the importance of this pathway in relation to SSG poor response in *Leishmania*.

Variants confined to CL-PR group are candidates for further study to investigate their functional role in causing poor response to SSG. We found 100 variants which could possibly result in pseudogenes (81 frame shifts, 2 stop lost and 17 stop gained) distributed among 71 genes in the Sri Lankan CL-PR group, while only 26 variants (24 frame shifts and 2 stop lost) were identified among 16 genes in the Sri Lankan CL wild type (Additional file 1: Table S22). Interestingly, a mitogen-activated protein kinase produces a pseudogene within our poor responders, down regulation of which has recently been found to be associated with antimony resistance in *Leishmania* field isolates [42].

## Discussion

The spectrum of clinical presentations in leishmaniasis as well as growing resistance to the limited number of effective drugs have resulted in many efforts to understand the parasite factors underlying these mechanisms. Developments in next generation sequencing technologies have facilitated in depth exploration of *Leishmania* parasites differing in these characteristics. This study focused on analysis and comparison of eight genomes of *Leishmania donovani* from Sri Lanka; isolated from six patients with CL and two patients with VL. Two of the CL isolates were from patients who demonstrated a poor response to antimonials.

Diversity in the genomes from the two phenotypes were suggested by our analysis based on chromosome and gene copy number variations as well as SNP distribution. Chromosome copy number/somy variations are common and well tolerated among *Leishmania* species [30, 43]. While analysis of chromosome somy showed a mostly disomic pattern in both groups there were some notable exceptions. A previous study of Sri Lankan *L. donovani* [14] describes chromosome 23 to be trisomic in CL-SL and VL-SL, but we observed a disomic pattern in chromosome 23 in both groups. The same study explains a disomic pattern in chromosome 13 and 20 in CL-SL and a trisomic pattern of same in VL-SL. In contrast, we observed a disomic pattern in chromosome 13 and 20 in both CL-SL and VL-SL. Monosomy is a relatively rare phenomenon in *Leishmania* with a few other reports of similar findings. For instance, in the analysis of two *Leishmania (Viannia) peruviana* isolates, chromosomes 1 to 5 in both were seen to be closer to monosomy [44] while Chromosome 2 was reported to be predominantly monosomic in a study of *Leishmania major* Friedlin (LmjF) strain [45]. Variation in somy per individual chromosome within the same species has been observed in *L. donovani* where phylogenetically close strains exhibited a different somy for 8 chromosomes including monosomy in chromosome 2 [6]. Chromosome 31 has been previously identified as supernumerary in all *Leishmania* species [43], generally tetrasomic in *L. donovani* [6]. Presence of aneuploidy is found to be common among *Leishmania* species while *L. infantum* and *L. donovani* have been previously identified to be the most aneuploid at the population level [6, 43, 45]. Aneuploidy is known to be closely mirrored at the transcript level thus affecting gene expression modulation [30, 46, 47].

We observed noninteger values for chromosome somy in several samples of CL and VL isolates. Such observations have been suggested to indicate mosaic aneuploidy; with varying levels of chromosomal content within a cell population [6, 30], which could result in intra-strain differences in parasite characteristics. Overall variability in ploidy in closely related isolates, as seen in the comparison of CL-SL and VL-SL could also be due to differences in exposure to in vitro conditions such as the number of passages [30], which however is an unlikely cause in the current isolates. Regional/segmental amplifications can occur through intrachromosomal rearrangements and have been reported in different laboratory settings [48, 49], as well as in natural parasite populations [7] as a response to drug pressure or other stressful conditions.

Our results also showed that the total number of arrays and the number of gene copies within an array varies among isolates of the same species which cause different phenotypes. Such increased gene content due to duplication may lead to an increase in the transcript levels of multicopy genes [43]. Among the genes with a significantly higher copy number in VL-SL compared to CL-SL were those involved in energy metabolism and amino acid metabolism. These potential functional differences may modulate immune responses by the host cells within which the parasites grow and survive, consequently contributing to distinct disease phenotypes. Striking differences in amino acid use and metabolism have been reported previously between *Leishmania* species associated with different phenotypes [50]. Several other genes with higher copy numbers in VL-SL, were those associated with virulent features favoring tropism for visceral organs. It is evident from these results that marked chromosome and gene copy number variations can be present within isolates of the same species, which may be adaptive virulence mechanisms.

The *A2* gene locus, which has been the focus of several studies exploring the parasite’s invasive characteristics during infection [14, 51, 52], was not seen to be differentially distributed among CL-SL and VL-SL groups in our study. This finding suggests that intra species variations resulting in different tropisms are likely to be influenced by many genes and their cumulative effects, as compared to phenotypic differences being determined by variations in a single gene locus or region. Transcriptional data from multiple isolates would add to the functional relevance of above observations.

The SNP based phylogenetic analysis suggested genetic diversity of *L. donovani* in Sri Lanka to be much higher than previously shown [7]. According to the phylogenetic tree, CL-SL and VL-SL strains have clearly different genetic origins and they might have established themselves much earlier than the ones in India and Nepal. Notably, the *L. infantum* branch seemed shorter than expected. This might have been caused by the fact that the reads were mapped to a *L. donovani* reference, and where *L. infantum* is very different from *L. donovani*, the reads that could not be mapped to the regions remained as gaps, likely leading to a shorter branch length.

The SNP analysis showed CL-SL and VL-SL to form two distinct groups with the CL-PR group also clustering separately. Non-synonymous SNPs present in coding regions are deemed particularly important because they could lead to changes at the protein level. Such variations unique to isolates from one phenotype may indicate functional differences which influence its characteristics. However, the distribution of non-synonymous SNPs among our isolates did not show major differences that could possibly explain the phenotypic differences. A previous phylogenomic study of clinical isolates of *L. donovani* from the Indian subcontinent showed Sri Lankan isolates to be closely related to the core group (ISC2–10) of samples as described in its [7] analysis. None of the unique SNPs or indels which could be used to identify each of these populations [53], as suggested subsequently as an alternative to whole genome sequencing for epidemiological purposes, were present in our isolates.

Drug resistance in *Leishmania* has often been related to copy number variations in specific genes although specific RNA levels have also been detected to change without affecting gene copy number [54–56]. We observed several genes associated with resistance to antimonials as well as some genes that can be attributed to vital biological processes such as parasite growth and survival, to be present in higher copy numbers in the poor responders to treatment (CL-PR). Similar associations were also seen with some genes with high counts of non-synonymous SNPs among our CL-PR group. The presence of amplifications at H-Locus, which has been implicated in drug resistance [47, 57], was more ambiguous with this being observed in CL-WT as well as VL-SL. Poor response to treatment in leishmaniasis can be attributed to many causes including variability in drug quality, dose, technique of administration and host factors. Thus, demonstration of true drug resistance of parasites requires confirmation with in vitro drug sensitivity testing. However, variations at parasite gene/genome level in the background of such clinical observations provide valuable information on possible candidates for further study.

## Conclusions

This comparative analysis of whole genome sequences attempted to elucidate genetic factors of virulence in *L. donovani* clinical isolates, largely responsible for an unusual presentation of cutaneous leishmaniasis. A range of genomic differences were observed predominantly at chromosome and gene level and to some extent in SNP distribution in the CL and VL isolates. Our results further indicate that aneuploidy is common and the pattern of inter-strain aneuploidy and intra-strain mosaicism vary within the CL and VL isolates of Sri Lankan *L. donovani,* possibly giving rise to differential expression of genes, ultimately leading to different disease tropisms.

Overall, the genes implicated are mostly those involved in growth and metabolic processes suggesting that adaptive virulence mechanisms may play a significant role in the pathogenesis by the different isolates. While we recognize that the lower number of VL isolates is likely to have affected the statistical comparisons, increasing the number of samples should be complemented by analysis at transcriptional and functional level to validate these findings. It is also known that the sequencing technology as well as the sequencing kits used can affect the subsequent analyses.

To our knowledge, this is the first genomic analysis of *L. donovani* strains demonstrating poor response to treatment in a cutaneous phenotype. In a background where the mechanisms of action of the widely used drugs still remain obscure, especially within a clinical setting, this information adds to the possible parasite genetic determinants of response to antimonials. The occurrence of H-locus amplification in our VL isolates which have not been exposed to antimonials highlights the need for further evaluation of these potential molecular markers.

The genomic differences identified in this study contribute to refining the understanding of molecular mechanisms underlying pathogenesis and drug responses of this parasite with known genome plasticity. While the findings favor the presence of different strains which may result in distinct phenotypes, host immune response determinants as well as sand fly interactions should be taken into consideration in interpreting the contribution of these genetic differences.

## Methods

### Sample collection

Six cutaneous leishmaniasis (CL-SL) and two visceral leishmaniasis (VL-SL) clinical isolates were obtained from Sri Lankan patients (Additional file 1: Table S1-(A)). All patients had no travel history out of the country and were residents of areas endemic for leishmaniasis.

The CL-SL clinical isolates were derived from aspirates or punch biopsies of skin lesions while the VL-SL clinical isolates were from bone marrow aspirates. Among the CL-SL isolates two were reported as poor responders (CL-PR) to SSG. Patients who did not demonstrate satisfactory healing after weekly intra-lesional SSG for 10 weeks were identified as poor responders in the local clinical setting, as per established criteria referred previously [58].

### Genomic DNA library preparation and sequencing

Genomic DNA was extracted from CL-SL and VL-SL clinical isolates to prepare paired-end DNA sequencing libraries of 500 bp fragments using Nextera DNA sequencing library preparation kits. 300 bp paired-end reads (Additional file 1: Table S1-(B)) were generated from these DNA sequencing libraries on an Illumina MiSeq platform at the Human Genetics Unit, Faculty of Medicine, University of Colombo, Sri Lanka.

### Preliminary analysis

The genome reference of *Leishmania donovani* BPK282A1 strain from Nepal obtained from GeneDB database (LdBPK282V1) [59] was used as the reference genome for the analysis. Sequencing reads of each CL-SL and VL-SL samples were analyzed with the use of FastQC tool [60] for base quality and overall GC content distribution. The paired-end reads from each sample were mapped to the *L. donovani* BPK282A1 reference using BWA-mem tool [61]. Mapped BAM files were cleaned, sorted, validated and duplicates were marked using Picard tool [62]. GATK toolkit [63] was used for further processing and analysis of the reads. Mapping and variant calling were repeated adhering to the same protocol against the recently generated reference for *L. donovani* on a Pacific Biosciences (PacBio) sequencing platform (LdBPK282V2) [30]. This new reference is more complete at repetitive regions but all public databases such as TriTrypDB [3] still maintain the previous version. We therefore performed the main analyses based on the genome reference LdBPK282V1.

### Chromosome and gene copy number analysis

Chromosome copy numbers/somy were calculated for each chromosome in each sample separately as described elsewhere [6, 7, 30]. Read depth for each position of chromosome was obtained using SAMtools [64]. Median read depth of each chromosome was divided by median of all chromosomal medians of a sample to get a raw somy value for a chromosome. A custom written python script “Chromo_median.py” was used to automate these calculations. This raw somy value was adjusted using the 4 neighboring chromosomes on each side and the chromosome itself. Adjusted somy value for a chromosome was calculated by dividing the median somy of these 9 chromosomes by the raw somy value of the chromosome [30] (Additional file 1: Table S2). Somy (S) was identified as monosomy, disomy, trisomy, tetrasomy and pentasomy as previously described by others as S < 1.5, 1.5 ≤ S < 2.5, 2.5 ≤ S < 3.5, 3.5 ≤ S < 4.5, 4.5 ≤ S < 5.5 respectively [6, 7, 30, 43]. Chromosome copy number data was saved in an 8 by 36 numeric matrix and similarity measures were calculated between the 8 *L. donovani* isolates and their aneuploidy profiles by VCFtools. This data was then used to generate a heat map with heatmap.2() function.

Gene copy number was calculated for each gene from each library as described previously [14, 43]. Read depth for each position of each gene on a chromosome was obtained using SAMtools. A bed file containing *L. donovani* gene positions was used to achieve this. A custom written python script “Gene_median.py” was used to obtain median read coverage per gene, and it was divided by the median coverage of the entire chromosome to obtain a normalized copy number for the gene. These values were used to calculate an average read depth per gene for the CL-SL and VL-SL groups and genes with an average read depth per gene > 0.5 were retained. A VL/CL ratio per gene was calculated based on these average values and genes with at least a two-fold change in copy number (either direction) between the Sri Lankan VL and CL isolates, were assumed to have biologically significant implications to their functions, and were used for downstream analysis. This set of genes was also evaluated for statistically significant copy number changes by applying Mann-Whitney U (MWU) test [65] with Benjamini and Hochberg false discovery rate (FDR) correction [66] for multiple comparisons. As the small number of samples limits the robustness of statistical testing, we defined each read as an observation point and used normalized read depth values of all positions within a gene as the data set, to take advantage of genome sequencing read information for the statistical comparison. Multicopy genes/tandem arrays, which are genes of more than one copy with the same OrthoMCL group ID and present on the same chromosome, were identified using OrthoMCL-DB [67]. Read coverage across chromosomal regions were visualized using Artemis tool [68] and Integrative Genomics Viewer [69].

### SNP and Indel analysis

SNPs and small indels were analyzed in detail in the three distinct phenotypes; CL-SL, VL-SL and CL-PR in comparison to the *L. donovani* reference. GATK’s HaplotypeCaller (HC) module was used in ERC GVCF mode to call SNPs and indels in each sample using minimum base quality (−mbq) of 21 and -stand_call_conf 30. HC produced .gvcf files were joined using GATK’s GenotypeGVCFs. The resultant multi-sample vcf file with raw variants were filtered for SNPs and indels respectively. These SNPs and indels were hard-filtered in order to extract high quality ones. QD < 2.0, FS > 60.0, MQ < 40, MQRankSum < − 12.5, ReadPosRankSum < − 8.0, SOR > 2.5 thresholds were used to filter SNPs and QD < 2.0, FS > 100.0, ReadPosRankSum < − 10.0 and SOR > 5.0 as thresholds used to filter indels. SNPs and indels with read-depth coverage (DP) < 10 and DP > 200 were excluded from analysis, as well as QUAL < 1500. Maximum DP was set 5–6 standard deviations from the mean coverage across all samples. Additionally, genotype fields were filtered for read-depth coverage (DP) < 8 and genotype quality (GQ) < 20. Filtered sites, genotypes and non-variants were all excluded from analysis. SNPs and indels in regions of first 3 kb and last 5 kb of each chromosome were excluded to remove potential low quality variants. Tantan tool [70] was used to remove variants in low complexity repeat regions.

In order to obtain a consistent set of variants for each group of CL-SL and VL-SL, and to minimize incorrect variant calling, we retained the variants present in at least half of the samples in each group. The resultant vcf files were used as input for SnpEff [71] to annotate the variants. Genes likely to be under strong selection were identified based on a ratio of non-synonymous SNP counts (P_N)_ to sum of synonymous (P_S)_ and non-synonymous (P_N)_ SNP counts above 0.5.

VCFtools [72] and a multi-sample vcf file were used to calculate a relatedness measure among the 8 *L. donovani* isolates based on SNP data and a similarity matrix was built up using R [73]. This matrix was used to generate a heat map using heatmap.2() function in gplots R package [74]. Bioconductor package SNPRelate [75] was used to remove SNPs in high linkage disequilibrium within our multi-sample vcf and to generate a pruned SNP set. This pruned SNP set was then used to carryout principal component analysis.

SNP allele heat maps were also created using the multi-sample vcf file with all 8 *L. donovani* isolates. Perl scripts were used to extract only the allele frequency from the vcf per each chromosome and finally a SNP allele frequency file per each chromosome was obtained. Allele frequency per bin (5 kb) was calculated for each chromosome using a perl script and heat maps per chromosome were generated using heatmap.2() function. Finally a single heat map was generated combining all files per chromosome.

### Phylogenetic analysis

We used a stringently filtered multi-sample vcf file consisting of our 8 Sri Lankan isolates and other key *Leishmania* strains discussed previously [7], to describe the genetic divergence levels among them by maximum-likelihood (ML) based phylogenetic inference. A fasta alignment file was generated from the multi-sample vcf file. RAxML [76] was used to find the best scoring maximum likelihood tree for the alignment using bootstrap convergence criteria with rapid bootstrapping and selecting the GTRGAMMA as the model of substitution. Visualizing and further analysis of ML tree was done by FigTree [77].

### Gene ontology

Gene ontology terms (GO terms) enriched within the set of differentially distributed genes among the two groups of CL-SL and VL-SL were identified by extracting the *Leishmania major* Friedlin (LmjF) orthologs corresponding to *L. donovani* genes and performing the GO enrichment analysis using TriTrypDB [3]. GO terms significantly overrepresented (*p*-value < 0.05) in the gene set for biological processes, molecular functions and cellular components were identified with Benjamini and Hochberg false discovery rate correction. Removal of the redundant GO terms in the set and generating scatterplots for the remaining terms were done using REVIGO, a web based semantic cluster algorithm [78].

## Additional files

Additional file 1: **Table S1-(A).** Summary of patient characteristics. **Table S1-(B).** Genome coverage and number of sequencing reads per sample **Table S2.** Calculation of chromosome somy. **Table S3.** Chromosome somy calls in Sri Lankan *L. donovani* six CL and two VL isolates. **Table S4.** Genes with highly variable copy numbers between Sri Lankan *L. donovani* CL and VL isolates. **Table S5.** Multicopy genes (tandem arrays) with highly variable copy numbers between Sri Lankan *L. donovani* CL and VL isolates. **Table S6.** Multicopy genes (tandem arrays) in Sri Lankan *L. donovani* CL isolates. **Table S7.** Multicopy genes (tandem arrays) in Sri Lankan *L. donovani* VL isolates. **Table S8.** Gene ontology enrichment in Sri Lankan *Leishmania donovani* CL and VL isolates based on genes identified with high copy numbers. **Table S9.** REVIGO analysis of GO ontology terms for genes with high copy numbers in Sri Lankan Cutaneous and Visceral *Leishmania donovani* populations **Table S10.** High SNP count genes in Sri Lankan *L. donovani* CL isolates. **Table S11.** High indel count genes in Sri Lankan *L. donovani* CL isolates. **Table S12.** High SNP count genes in Sri Lankan *L. donovani* VL isolates. **Table S13.** High indel count genes in Sri Lankan *L. donovani* VL isolates. **Table S14.** Predicted pseudogenes among *L. donovani* CL-SL and VL-SL isolates. **Table S15.** Genes under strong selection among the high SNP count genes in Sri Lankan *L. donovani* CL isolates. **Table S16.** Genes under strong selection among the high SNP count genes in Sri Lankan *L. donovani* VL isolates. **Table S17.** Genes with highly variable copy numbers between Sri Lankan *L. donovani* CL wild type and CL poor responders to sodium stibogluconate. **Table S18.** Multicopy genes (tandem arrays) in Sri Lankan *L. donovani* CL wild type. **Table S19.** Multicopy genes (tandem arrays) in Sri Lankan *L. donovani* CL poor responders to sodium stibogluconate. **Table S20.** Multicopy genes (tandem arrays) with highly variable copy numbers between Sri Lankan *L. donovani* CL wild type and CL poor responders to sodium stibogluconate. **Table S21.** Top genes with non-synonymous unique SNPs in Sri Lankan *L. donovani* CL poor responders to SSG. **Table S22.** Predicted pseudogenes and their functions in *L. donovani* CL wild type and CL poor responders to sodium stibogluconate. (XLSX 961 kb)

Additional file 2: **Figure S1.** Read coverage (based on all the positions) across each chromosome of Sri Lankan *L. donovani* CL isolate L2339. (PDF 1585 kb)

Additional file 3: **Figure S2.** Read coverage (based on all the positions) across each chromosome of Sri Lankan *L. donovani* VL isolate VL38. (PDF 1587 kb)

Additional file 4: **Figure S3.** Comparison of read coverage across the *A2* region in chromosome 22 of six CL-SL and two VL-SL clinical isolates. (PDF 62 kb)

Additional file 5: **Figure S4.** Two frameshift mutations on trypanothione reductase gene (LdBPK_050350) of Sri Lankan *L. donovani*. These two frameshifts are present in all the CL-SL isolates except R5_S5 and VL-SL isolates have intact copies. Frameshift at position Ld05_v01s1:104041 (left) is an insertion changing the reference ‘C’ allele to a ‘CTG’. The other frameshift is a deletion at position Ld05_v01s1:104042 (right) and it changes the reference ‘CAG’ allele in to a ‘C’. Both frameshifts are covered by more than 65% of the reads at both positions in all the 5 CL-SL isolates. Details were viewed in IGV. (PNG 23 kb)

Additional file 6: **Figure S5.** Heat map showing the SNP allele frequency distribution across chromosomes of Sri Lankan *L. donovani* isolates. Allele frequencies for 5 kb bins of all chromosomes from 1 to 36 from the top are on the y-axis and *L. donovani* CL and VL isolates are listed along the bottom x-axis. The colour bar represents the phenotype of isolate. Red-CL-SL and Black-VL-SL. The colour key with histogram represents the allele frequency distribution. (PNG 77 kb)

## Abbreviations

CDS: Coding DNA sequence
CL: Cutaneous leishmaniasis
CL-PR: Cutaneous leishmaniasis poor responders to SSG
CL-SL: Cutaneous leishmniasis Sri Lanka
CL-WT: Cutaneous leishmaniasis wild type
GO: Gene ontology
*LdAQP1*: *Leishmania donovani* aquaglyceroporin 1
LmjF: *Leishmania major* Friedlin
*MAPKs*: Mitogen-activated protein kinases
MWU: Mann-Whitny U test
S: Chromosome somy
SNP: Single nucleotide polymorphism
SSG: Sodium stibogluconate
VL: Visceral leishmaniasis
VL-SL: Visceral leishmaniasis Sri Lanka
