# Supplementary material for: Genomic insights into virulence mechanisms of Leishmania donovani: evidence from an atypical strain
Source: BMC Genomics. 2018 Nov 28;19:843. doi: 10.1186/s12864-018-5271-z (PMC6262978; doi:10.1186/s12864-018-5271-z)
Supplement: Supplementary file 3 — Figure S2. Read coverage (based on all the positions) across each chromosome of Sri Lankan L. donovani VL isolate VL38. (PDF 1587 kb) [file 12864_2018_5271_MOESM3_ESM.pdf]

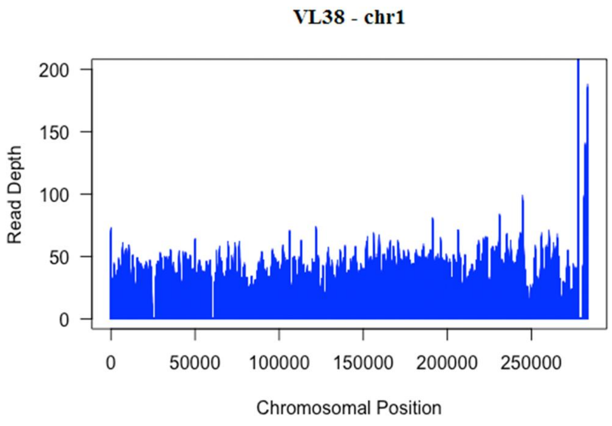
VL38 - chr1
Read Depth
0
50
100
150
200
0
50000
100000
150000
200000
250000
Chromosomal Position

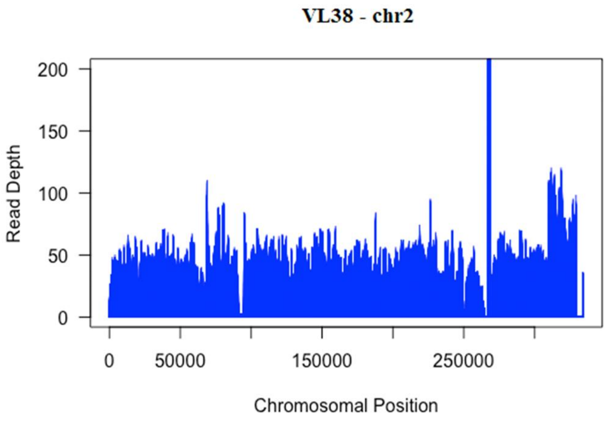
VL38 - chr2
Read Depth
0
50
100
150
200
0
50000
150000
250000
Chromosomal Position

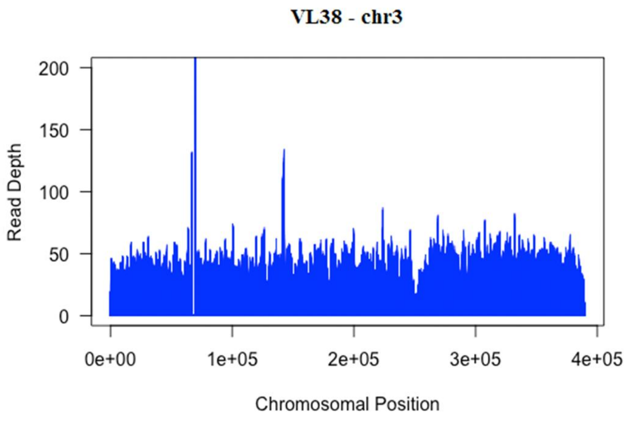
VL38 - chr3
Read Depth
0
50
100
150
200
0e+00
1e+05
2e+05
3e+05
4e+05
Chromosomal Position

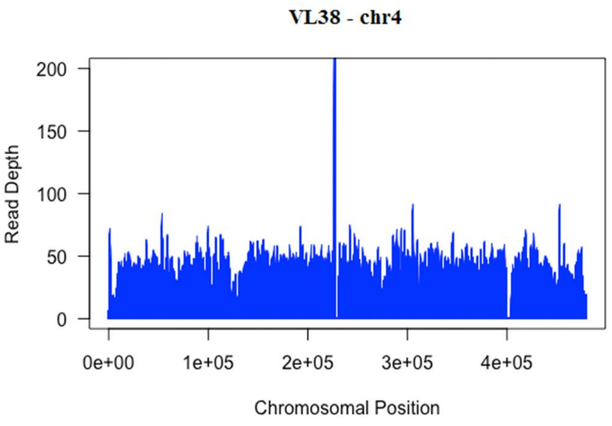
VL38 - chr4
Read Depth
0
50
100
150
200
0e+00
1e+05
2e+05
3e+05
4e+05
Chromosomal Position

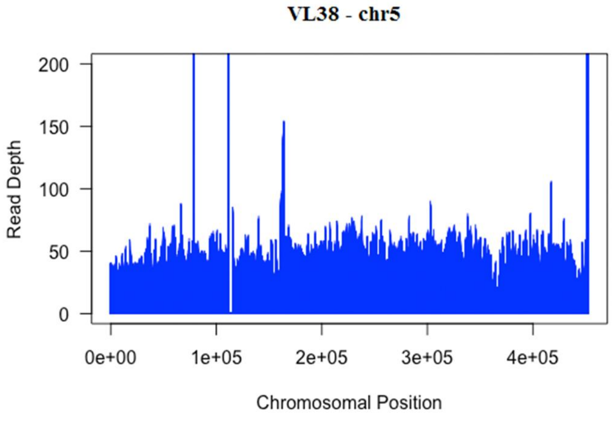
VL38 - chr5
Read Depth
0
50
100
150
200
0e+00
1e+05
2e+05
3e+05
4e+05
Chromosomal Position

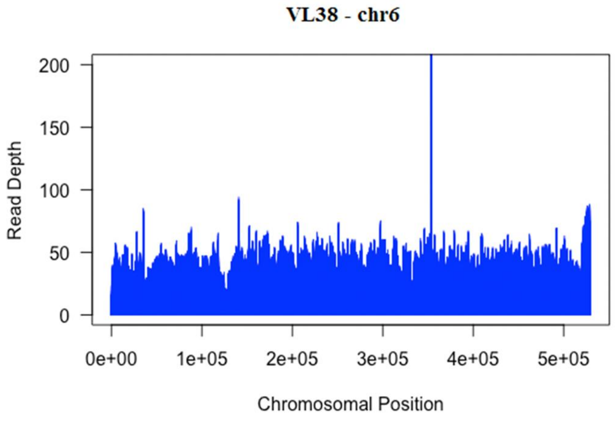
VL38 - chr6
Read Depth
0
50
100
150
200
0e+00
1e+05
2e+05
3e+05
4e+05
5e+05
Chromosomal Position

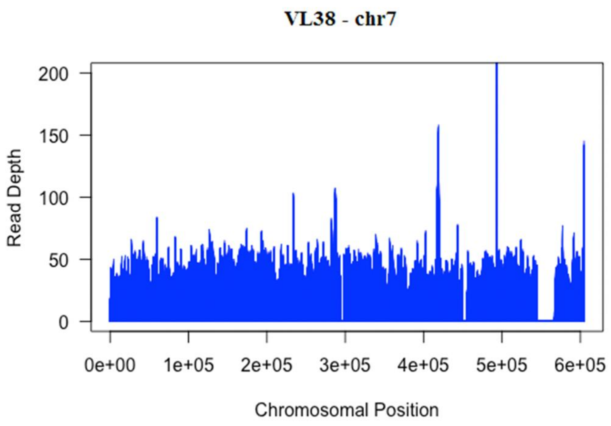
VL38 - chr7
Read Depth
0
50
100
150
200
0e+00
1e+05
2e+05
3e+05
4e+05
5e+05
6e+05
Chromosomal Position

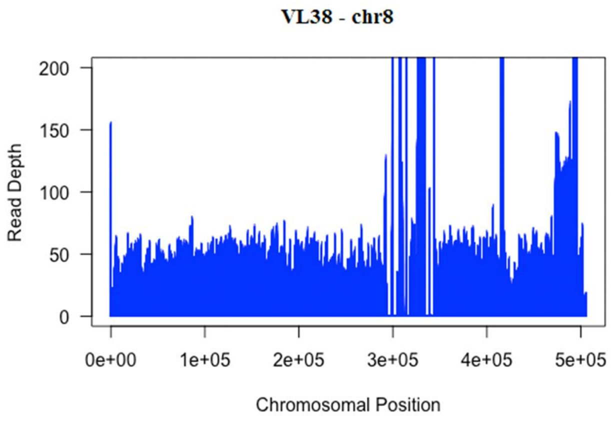
VL38 - chr8
Read Depth
0
50
100
150
200
0e+00
1e+05
2e+05
3e+05
4e+05
5e+05
Chromosomal Position

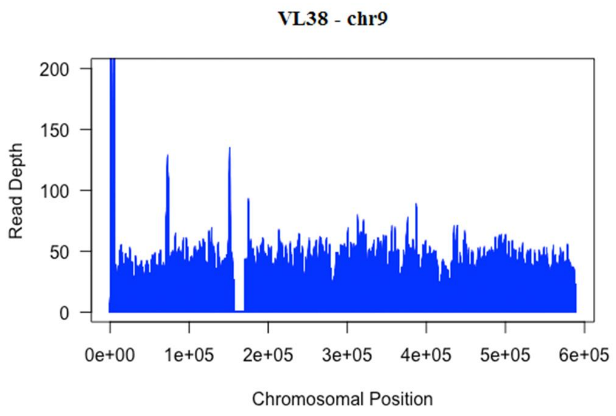
VL38 - chr9
Read Depth
0
50
100
150
200
0e+00
1e+05
2e+05
3e+05
4e+05
5e+05
6e+05
Chromosomal Position

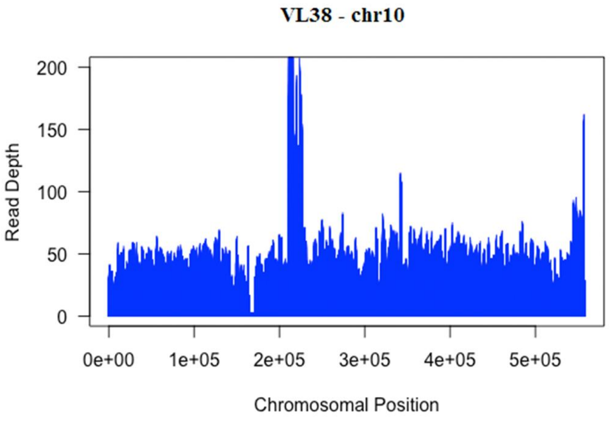
VL38 - chr10
Read Depth
0
50
100
150
200
0e+00
1e+05
2e+05
3e+05
4e+05
5e+05
Chromosomal Position

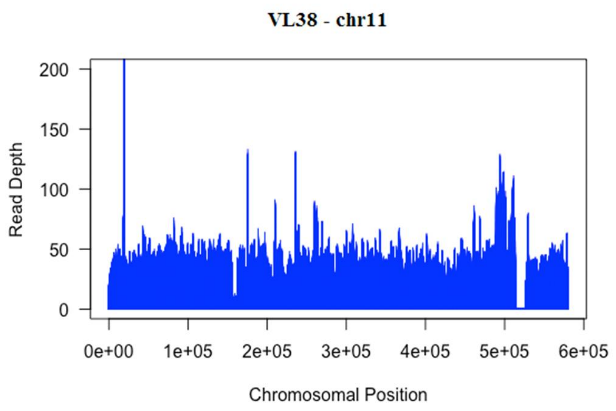
VL38 - chr11
Read Depth
0
50
100
150
200
0e+00
1e+05
2e+05
3e+05
4e+05
5e+05
6e+05
Chromosomal Position

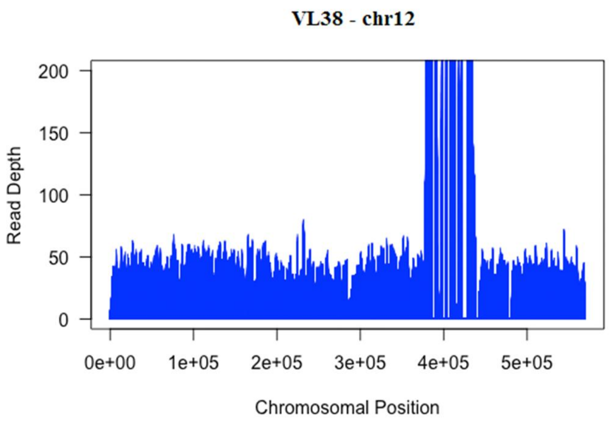
VL38 - chr12
Read Depth
0
50
100
150
200
0e+00
1e+05
2e+05
3e+05
4e+05
5e+05
Chromosomal Position

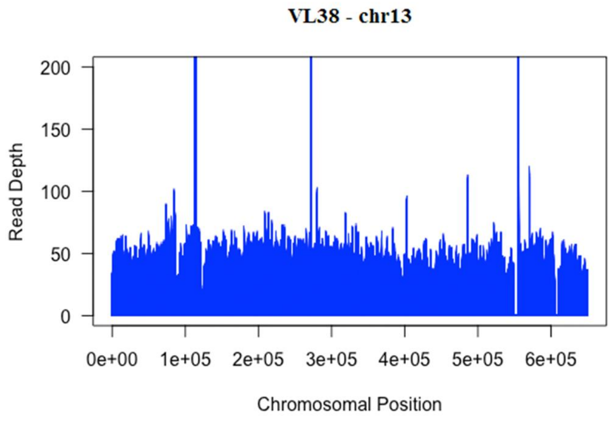
VL38 - chr13
Read Depth
Chromosomal Position
0 50 100 150 200
0e+00 1e+05 2e+05 3e+05 4e+05 5e+05 6e+05

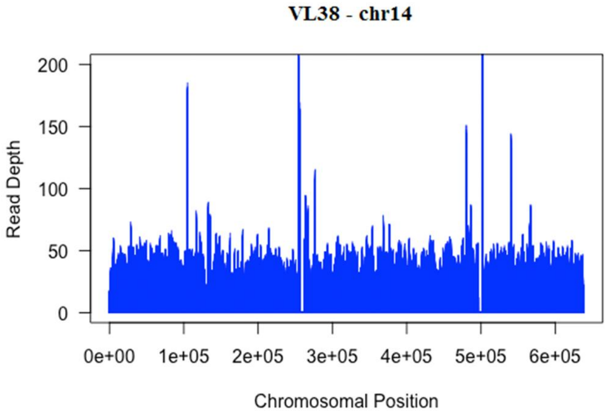
VL38 - chr14
Read Depth
Chromosomal Position
0 50 100 150 200
0e+00 1e+05 2e+05 3e+05 4e+05 5e+05 6e+05

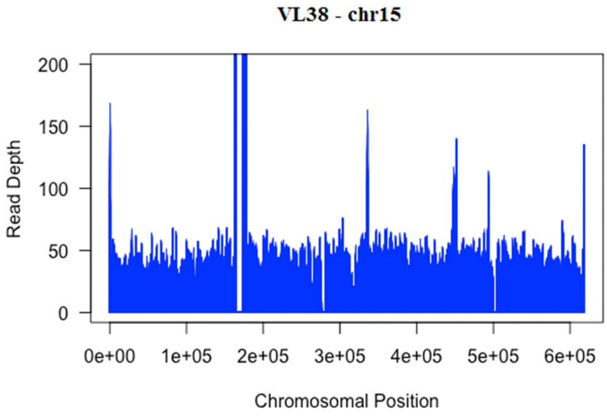
VL38 - chr15
Read Depth
Chromosomal Position
0 50 100 150 200
0e+00 1e+05 2e+05 3e+05 4e+05 5e+05 6e+05

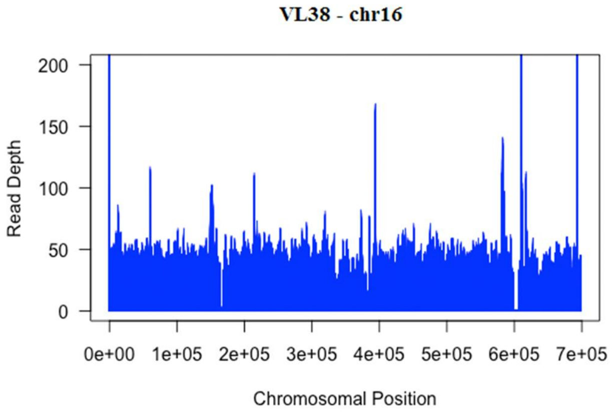
VL38 - chr16
Read Depth
Chromosomal Position
0 50 100 150 200
0e+00 1e+05 2e+05 3e+05 4e+05 5e+05 6e+05 7e+05

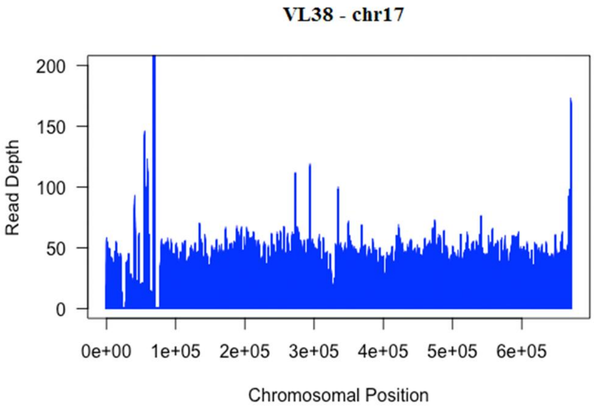
VL38 - chr17
Read Depth
Chromosomal Position
0 50 100 150 200
0e+00 1e+05 2e+05 3e+05 4e+05 5e+05 6e+05

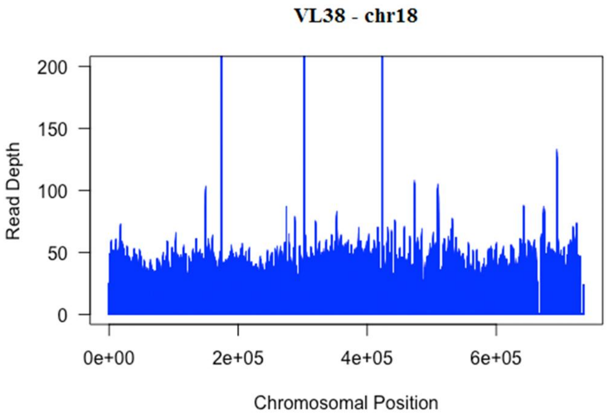
VL38 - chr18
Read Depth
Chromosomal Position
0 50 100 150 200
0e+00 2e+05 4e+05 6e+05

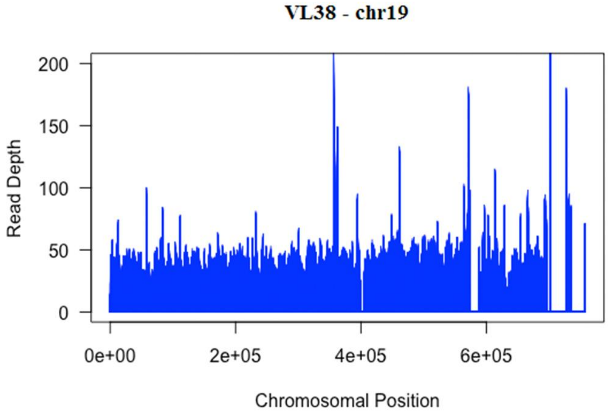
VL38 - chr19
Read Depth
0
50
100
150
200
0e+00
2e+05
4e+05
6e+05
Chromosomal Position

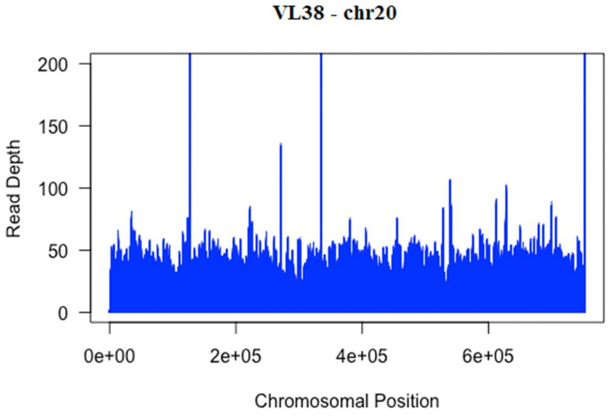
VL38 - chr20
Read Depth
0
50
100
150
200
0e+00
2e+05
4e+05
6e+05
Chromosomal Position

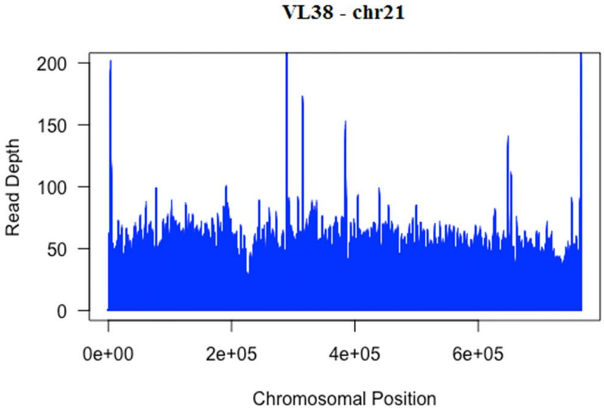
VL38 - chr21
Read Depth
0
50
100
150
200
0e+00
2e+05
4e+05
6e+05
Chromosomal Position

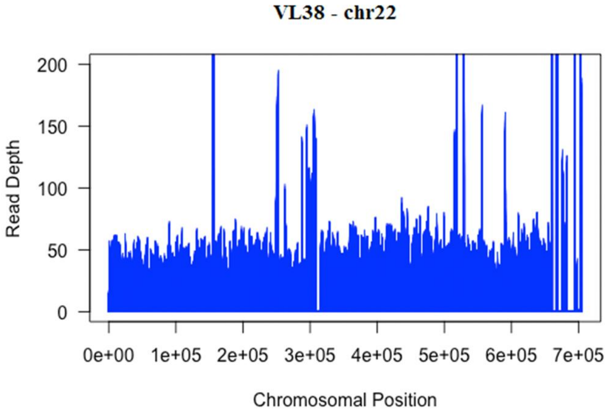
VL38 - chr22
Read Depth
0
50
100
150
200
0e+00
1e+05
2e+05
3e+05
4e+05
5e+05
6e+05
7e+05
Chromosomal Position

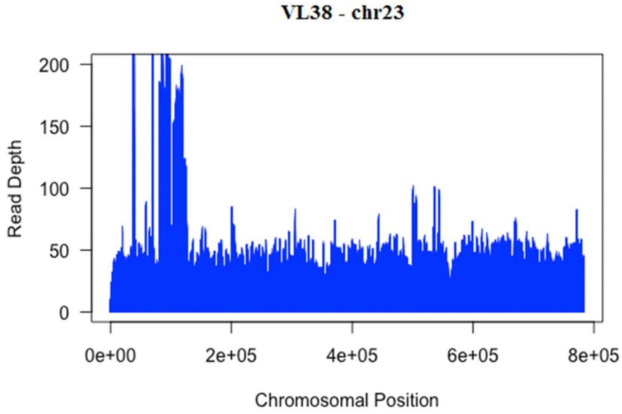
VL38 - chr23
Read Depth
0
50
100
150
200
0e+00
2e+05
4e+05
6e+05
8e+05
Chromosomal Position

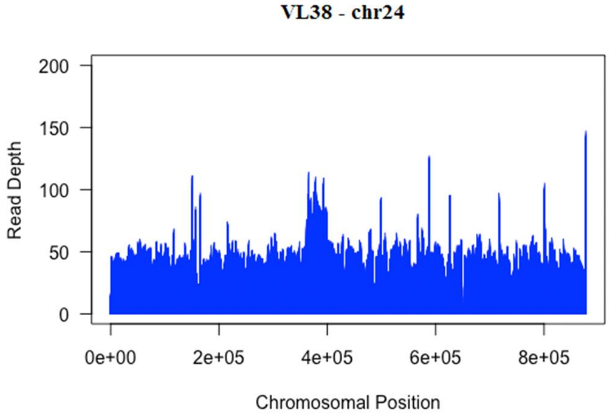
VL38 - chr24
Read Depth
0
50
100
150
200
0e+00
2e+05
4e+05
6e+05
8e+05
Chromosomal Position

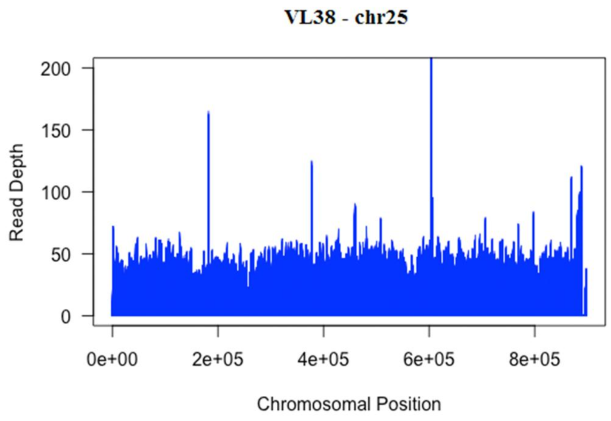
VL38 - chr25
200
150
100
50
0
Read Depth
0e+00
2e+05
4e+05
6e+05
8e+05
Chromosomal Position

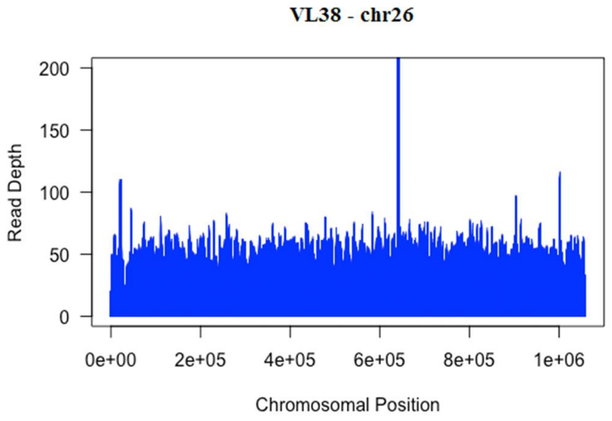
VL38 - chr26
200
150
100
50
0
Read Depth
0e+00
2e+05
4e+05
6e+05
8e+05
1e+06
Chromosomal Position

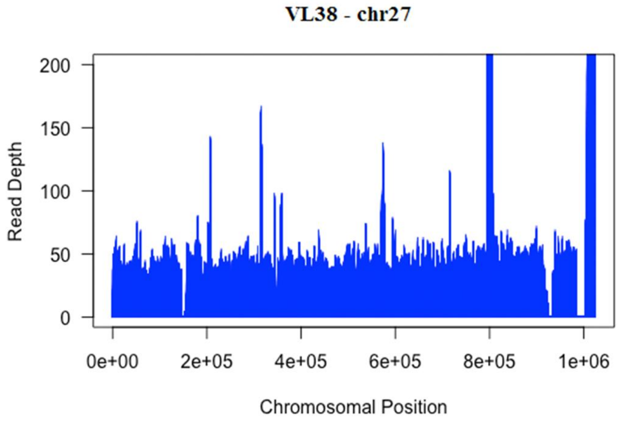
VL38 - chr27
200
150
100
50
0
Read Depth
0e+00
2e+05
4e+05
6e+05
8e+05
1e+06
Chromosomal Position

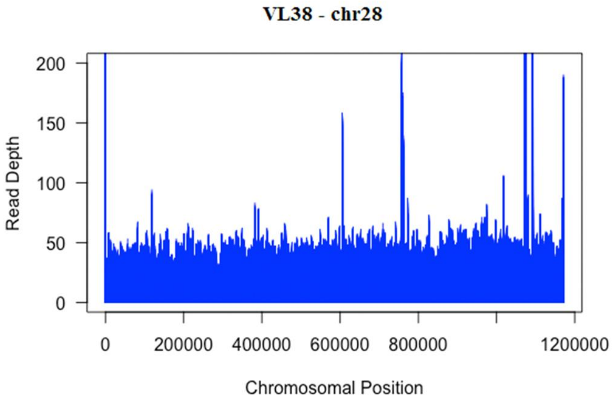
VL38 - chr28
200
150
100
50
0
Read Depth
0
200000
400000
600000
800000
1200000
Chromosomal Position

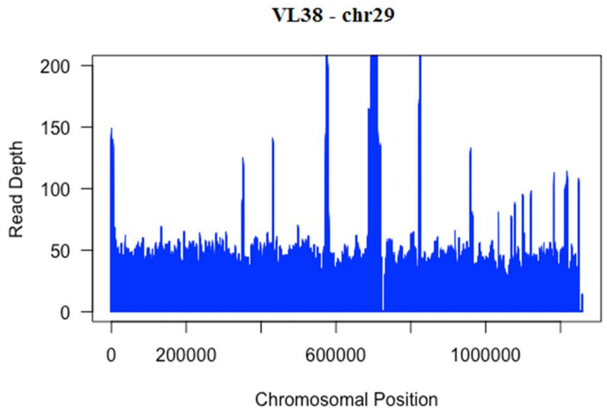
VL38 - chr29
200
150
100
50
0
Read Depth
0
200000
600000
1000000
Chromosomal Position

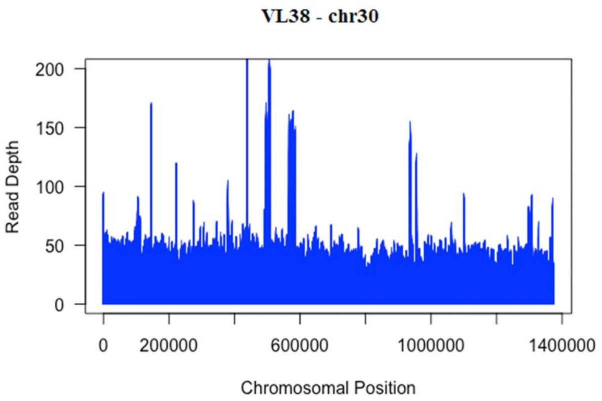
VL38 - chr30
200
150
100
50
0
Read Depth
0
200000
600000
1000000
1400000
Chromosomal Position

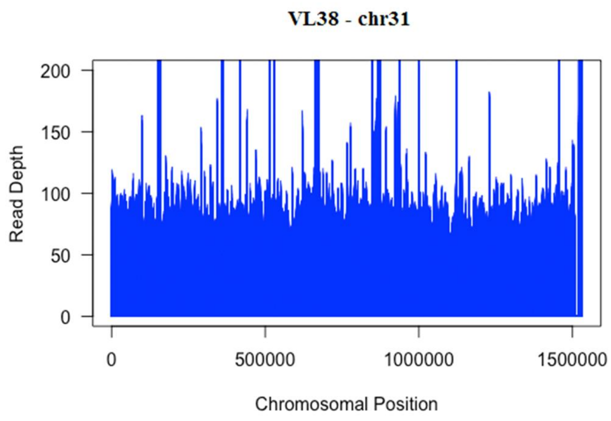
VL38 - chr31
Read Depth
200
150
100
50
0
0
500000
1000000
1500000
Chromosomal Position

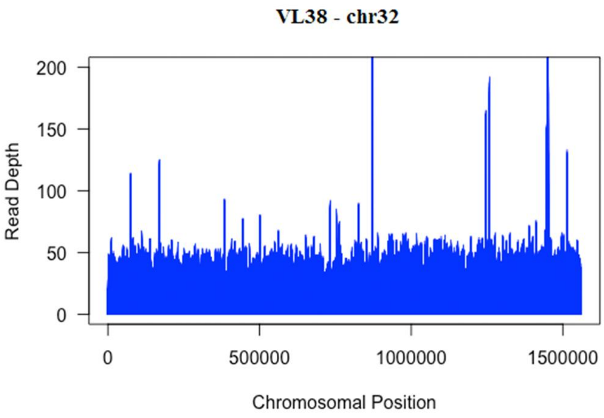
VL38 - chr32
Read Depth
200
150
100
50
0
0
500000
1000000
1500000
Chromosomal Position

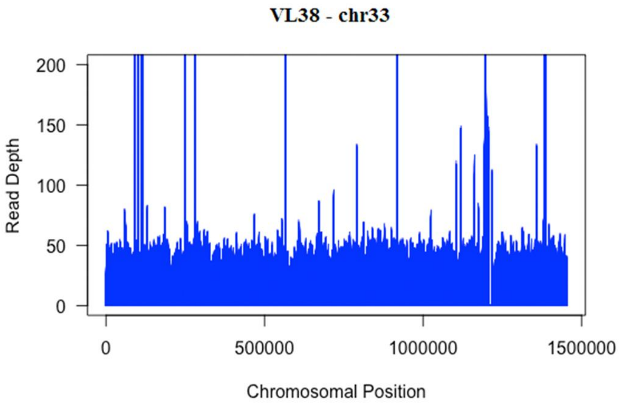
VL38 - chr33
Read Depth
200
150
100
50
0
0
500000
1000000
1500000
Chromosomal Position

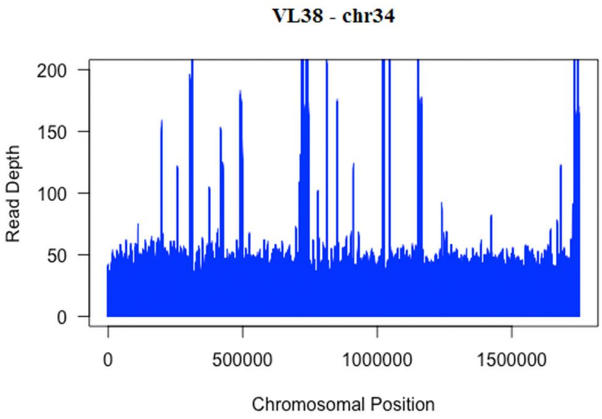
VL38 - chr34
Read Depth
200
150
100
50
0
0
500000
1000000
1500000
Chromosomal Position

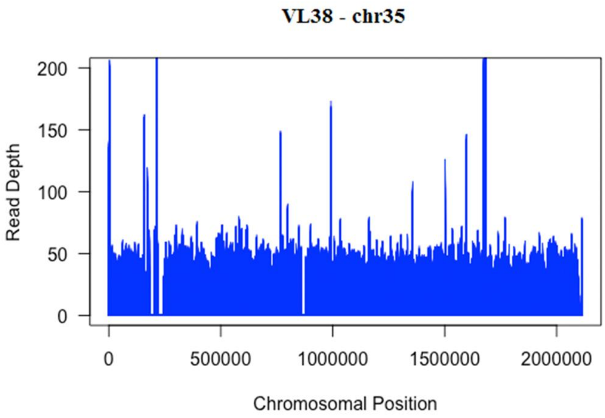
VL38 - chr35
Read Depth
200
150
100
50
0
0
500000
1000000
1500000
2000000
Chromosomal Position

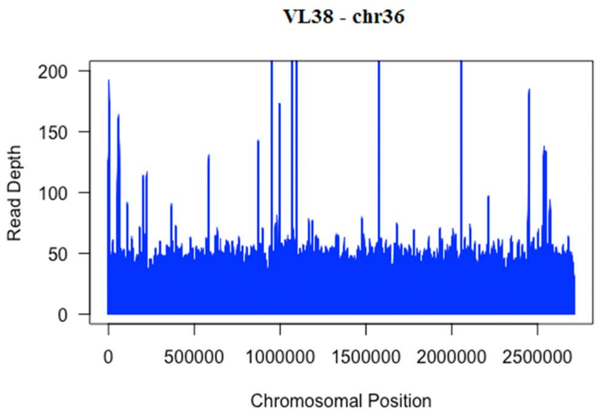
VL38 - chr36
Read Depth
200
150
100
50
0
0
500000
1000000
1500000
2000000
2500000
Chromosomal Position
