# Supplementary material for: Genomic insights into virulence mechanisms of Leishmania donovani: evidence from an atypical strain
Source: BMC Genomics. 2018 Nov 28;19:843. doi: 10.1186/s12864-018-5271-z (PMC6262978; doi:10.1186/s12864-018-5271-z)
Supplement: Supplementary file 4 — Figure S3. Comparison of read coverage across the A2 region in chromosome 22 of six CL-SL and two VL-SL clinical isolates. (PDF 62 kb) [file 12864_2018_5271_MOESM4_ESM.pdf]

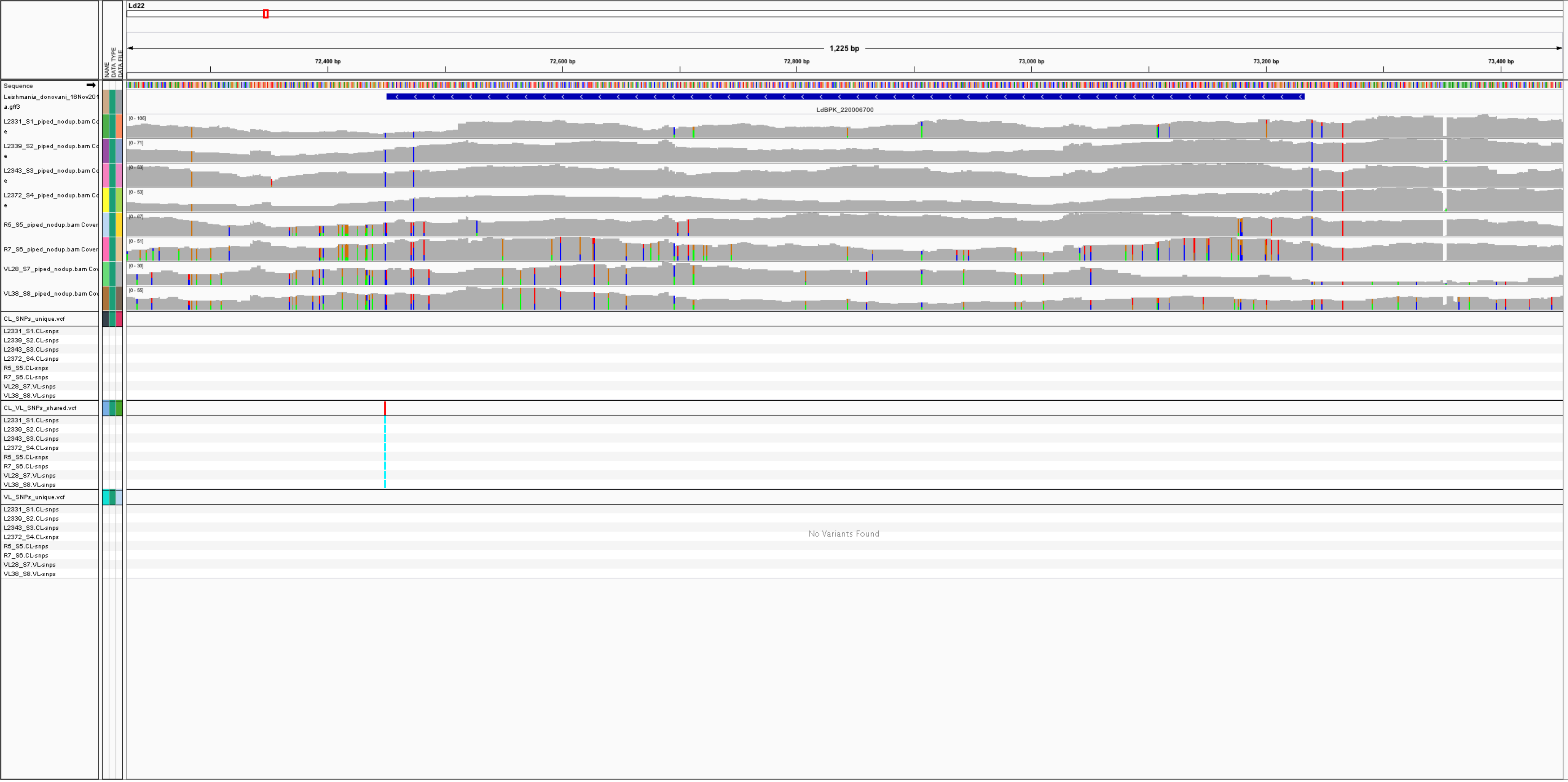

Figure S1: Comparison of read coverage across the A2 region (LdBPK_220670, LdBPK_220006700 in PacBio) in chromosome 23 of six CL-SL and two VL-SL clinical isolates. Reads were aligned to the new PacBio sequence of *L. donovani*. Only one SNP shared among the two groups of isolates was identified. Viewed using the IGV tool.
