# Supplementary figures and images for: Genomic insights into virulence mechanisms of Leishmania donovani: evidence from an atypical strain
Source: BMC Genomics. 2018 Nov 28;19:843. doi: 10.1186/s12864-018-5271-z (PMC6262978; doi:10.1186/s12864-018-5271-z)

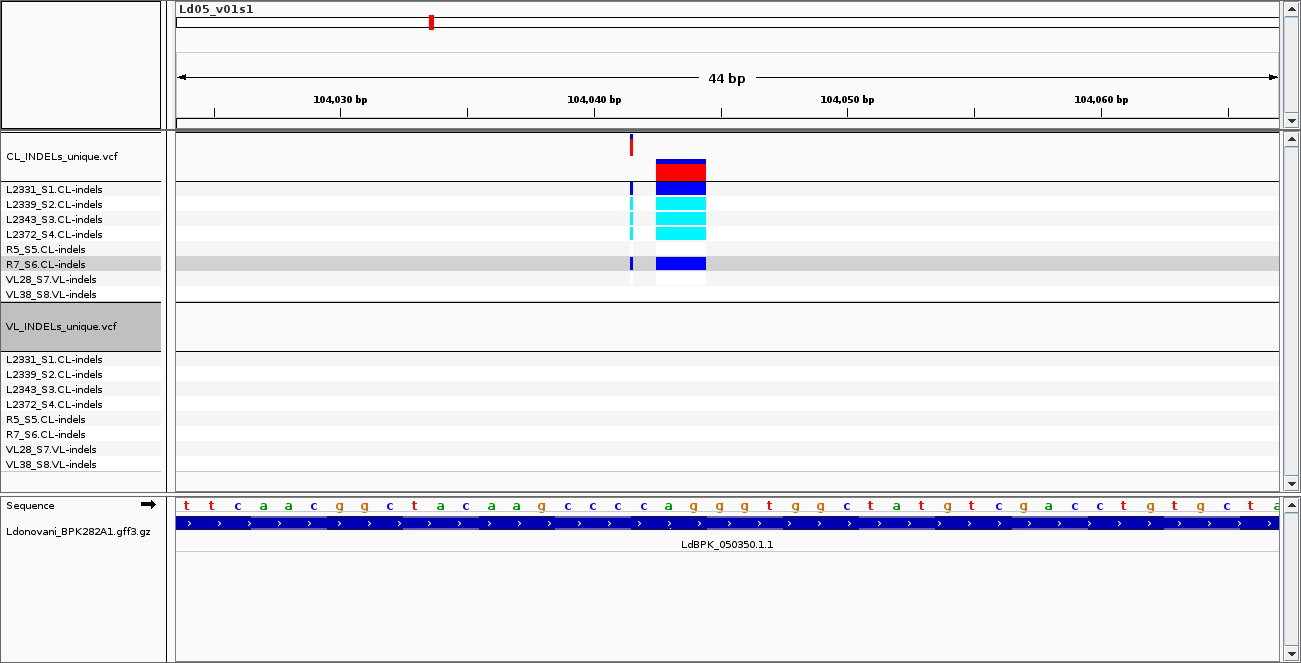

Supplement: Supplementary file 5 — Figure S4. Two frameshift mutations on trypanothione reductase gene (LdBPK_050350) of Sri Lankan L. donovani. These two frameshifts are present in all the CL-SL isolates except R5_S5 and VL-SL isolates have intact copies. Frameshift at position Ld05_v01s1:104041 (left) is an insertion changing the reference ‘C’ allele to a ‘CTG’. The other frameshift is a deletion at position Ld05_v01s1:104042 (right) and it changes the reference ‘CAG’ allele in to a ‘C’. Both frameshifts are covered by more than 65% of the reads at both positions in all the 5 CL-SL isolates. Details were viewed in IGV. (PNG 23 kb) [file 12864_2018_5271_MOESM5_ESM.png]

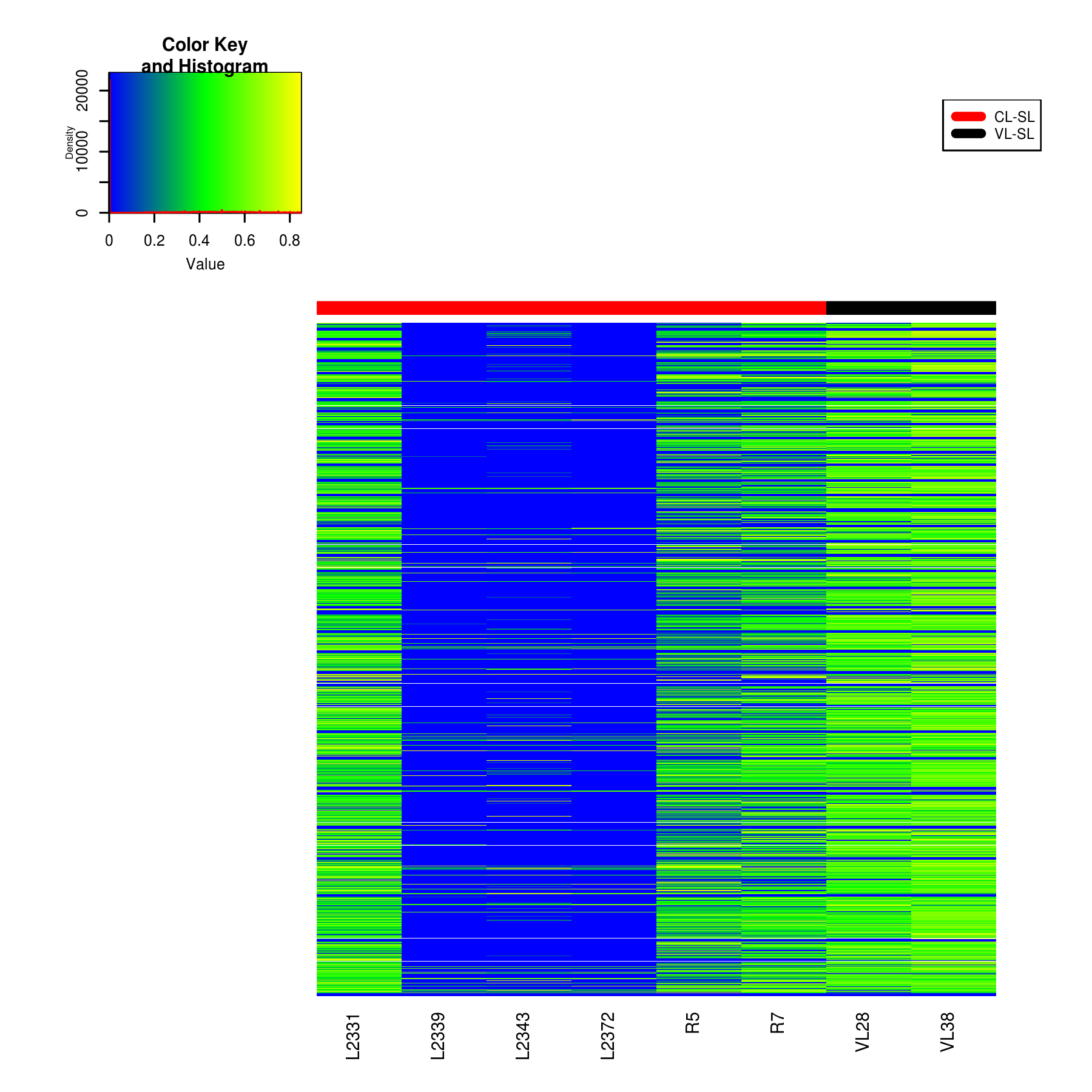

Supplement: Supplementary file 6 — Figure S5. Heat map showing the SNP allele frequency distribution across chromosomes of Sri Lankan L. donovani isolates. Allele frequencies for 5 kb bins of all chromosomes from 1 to 36 from the top are on the y-axis and L. donovani CL and VL isolates are listed along the bottom x-axis. The colour bar represents the phenotype of isolate. Red-CL-SL and Black-VL-SL. The colour key with histogram represents the allele frequency distribution. (PNG 77 kb) [file 12864_2018_5271_MOESM6_ESM.png]
